# Impact of Natural Language Processing models on diagnosis and decision-making in healthcare, business, education, and sports: a review

**DOI:** 10.3389/frai.2025.1706369

**Published:** 2026-02-05

**Authors:** Aryan Choudhary, Sireesha Pamidimokkala, Krithiga R., Bhavadharini R. M.

**Affiliations:** School of Computer Science and Engineering, Vellore Institute of Technology, Chennai, Tamil Nadu, India

**Keywords:** Natural Language Processing (NLP), BERT, Customer Relationship Management (CRM), Named Entity Recognition (NER), Business Process Modeling (BPM), Electronic Health Records (EHR), Convolutional Neural Networks (CNN)

## Abstract

Natural Language Processing (NLP) has an influence on almost every field nowadays, such as business, healthcare, and sports, by making advanced interactions with human language and providing analytics. In the field of business, NLP has been a revolution, bettering customer service with the help of advanced chatbots, sentiment analysis, and automation in generating content, which enhances efficiency, personalization, and most importantly, decision-making. In healthcare, NLP is of crucial importance in decoding unstructured data like of medical records, supporting diagnostic accuracy, and making patient communication smoother, leading to better outcomes and improving efficiency. When it comes to sports, NLP provides critical insights through performance analytics, media content interpretation, and improved fan engagement, transforming data to utilize it for our advantage. The aim of this review is to systematically evaluate NLP's effectiveness across these sectors, address possible and existing challenges, and propose approaches for future research. Through the integration of case studies and performance assessments, we seek to clearly explain how NLP promotes innovation, resolves complex issues, and has made contributions to advancing new heights in these domains.

## 1 Introduction

Natural Language Processing (NLP) merges the interdisciplinary fields of computer science, artificial intelligence, and human linguistics to enable the development of systems that can understand, interpret, and generate human language. Thus this methodology is used to facilitate easy and accurate human computer interaction. Reshamwala et al. (2013) explain that NLP operates using phonology, speech sounds, morphology, syntax, and semantics on different levels. Early research in the fields of NLP and Machine learning dates back to the 1940s, focusing more on the Russian and English languages. The systems of this era focused on the question-and-answer type of systems which led to the development of models like LUNAR, and BASEBALL. This was followed by the usage of statistical language modeling and computational grammar theory. Khurana et al. (2023) in their extensive review of NLP, noted that by the 2000s, with the introduction of neural networks, sequence-to-sequence models like Convolutional Neural Networks, and recurrent neural networks (RNNs), such as Long Short-Term Memory (LSTM) and Gated Recurrent Units (GRU) were being put to practical use. Recently, with the development of attention mechanisms and transformers, models like BERT have enhanced contextual understanding and long-term dependency learning.

Parallely, the Third Industrial Revolution sparked the widespread use of computers in businesses. The inventions of transistors, microchips, and personal computers made it possible for businesses to shift to computers and technology of tasks like data processing, communication and automation. Berisha-Shaqiri (2014) explains how the revolution in information and communication technology gave humongous opportunities for corporations to reach more customers, introduce new products and services quickly, and collaborate with suppliers and business partners from all over the world.

In the healthcare industry, there has been a rapid development of information data. Communication technologies have addressed numerous challenges of safety of patients, managing diets, telemedicine, and digital imaging. Burney et al. (2010) illustrate many technologies like telemedicine, barcode systems, identification of radio frequency (RFID), and Clinical Decision Support Systems (CDSS) striving to improve patient safety, streamlining administration, and optimization of dietary management. Not only we are provided with a cost effective method of healthcare management, but also have a new sphere of Medical Care opened, which is more accurate, efficient, and robust than traditional methods.

Even in professional sports, there are huge and varied datasets that come with challenges for timely analysis. AI can be helpful, but sports experts most of the time lack the skill of Artificial Intelligence (AI). The paper by Pavitt et al. (2021) explores the accessibility of AI services, specially Natural Language Processing (NLP) and Conversational Interfaces, and how they assist in data analysis. The tools are used to find intuitive ways for sports professionals in exploring data and gaining insights without any advanced AI knowledge, enhancing decision-making and saving time. A trial was done with Leatherhead Football Club, a semi-professional team, demonstrating the potential benefits of making AI and analytics accessible to every level where resources are limited.

NLP is applied to education, transforming learning experiences and efficiencies in instruction. Examples of these applications are GPT models that assist in student comprehension, provide detailed answers, or even solves math problems by finding pertinent equations and classifying questions that students present at the appropriate cognitive level; though there is still scope for common sense and consistency in applying the cognitive levels. NLP-based applications, such as ChatGPT, are also increasingly used for text generation, editing, and evaluation. In this respect, opportunities and risks are presented for education. On the one hand, it democratizes access to learning assistance and streamlines processes in areas such as computer science and IT security, while risks arise from cheating on assignments and compromising assessment integrity. Once this is actualized, for example, effective adaptation strategies such as fostering ethical awareness will become imperative in harnessing the potential of NLP in educational settings.

**Figure 1:**
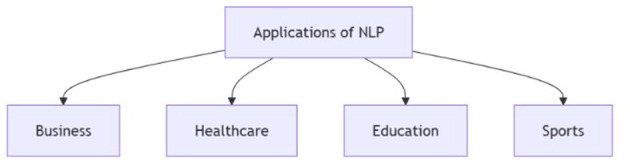
Applications of NLP.

Thus, NLP analysis in business, healthcare, sport, and education can be seen in a large extent as an area that bridges human language and machine code, providing intuitive access to difficult technologies in these sectors. Figure 1 shows the applications in NLP. This paper will explore the multifaceted applications of NLP and how organizations use it to seize data and enhance their operations and services.

## 2 NLP in business

### 2.1 Customer interaction and sentiment analysis

Increasingly, businesses have utilized Natural Language Processing (NLP) to help advance automation, create workflow efficiencies, facilitate data analytics, and improve Customer Relationship Management (CRM). As organizations increasingly adopt computational linguistics to enhance their IT ecosystems, NLP systems allow for more superior human-computer interaction and facilitate data-driven decision-making. Table 1 shows the micro application of NLP in business.

**Table 1:** Micro-applications of NLP in business.

**Domain**	**Micro-applications**
Business	Sentiment analysis
	Customer Relationship Management (CRM)
	Business Process Modeling (BPM)
	Document processing
	Business Intelligence (BI)
	Construction information management
	IoT and AI integration
	Ethically governed AI

Kang et al. (2020) reviewed management literature on NLP and noted there wasn't an encompassing recommendation on its applications. The authors reviewed papers from the UT Dallas List of 24 Leading Business Journals, focusing on consultations of relevant toolkit lists, data analysis, homework assignments, procedures and managerial factors related to NLP in business disciplines.

A leading use case of NLP in business is sentiment analysis–where NLP technologies attempt to project stock market tendencies and consumer behavior by analyzing social media and news data or reports. Bahja (2020), made pointed mention of NLP's contributions to e-governance via public engagement techniques and the applicability of machine learning in citizen feedback-based decision making. These capabilities are widely available in CRM systems to improve customer satisfaction and retention efforts.

Olujimi and Ade-Ibijola (2023) provided a review of 73 previous scholarly articles investigating chatbots and digital assistants utilized in banking, education, and the legal sector. The authors explain how NLP techniques (e.g., word sense disambiguation techniques and neural machine translation) can help optimize for clear case information, clarify ambiguous terminology, and query meticulous meaning from a corpus to improve automated customer support.

E-businesses also rely upon NLP-based chatbots that allow for conversations that are like those that occur with humans. Also, voice assistants such as Amazon Alexa and Google Assistant utilize NLP to generate responses based on contexts, while Salesforce's Einstein integrates NLP to develop real-time recommendations that improve the user experience and user loyalty.

This paper focuses on the most significant opportunities and challenges of remote work, adopted by digital technologies during the COVID-19 pandemic. The study by Jose Ramon et al. (2022) analyzed user-generated content on Twitter by using data-mining techniques, namely sentiment analysis (with TextBlob) and topic modeling (with Latent Dirichlet Allocation), and discovered 11 topics: 3 negative (virtual health, privacy concerns, stress), 4 positive (work-life balance, reduced stress, future, engagement), and 3 neutral (new technologies, sustainability, technology issues). The main opportunities are improved work-life balance, increased environmental sustainability, and reduced stress in commuting. The challenges involved are issues of privacy, the most optimal digital platforms, mental health, and adequacy of equipment. Results above indicate that companies should strive to develop enabling remote work strategies supporting engagement with stress, privacy, and technical issues addressed for successful telework.

### 2.2 Business intelligence and data analytics

NLP adds value to Business Intelligence (BI) by deriving insights from unstructured data such as potential customer feedback, market research, and social media. Traditional keyword searches are limited by context ambiguity; therefore, text mining and semantic web technologies (Gupta and Narang, 2012) have been used to improve predictive analysis and knowledge discovery.

**Figure 2:**
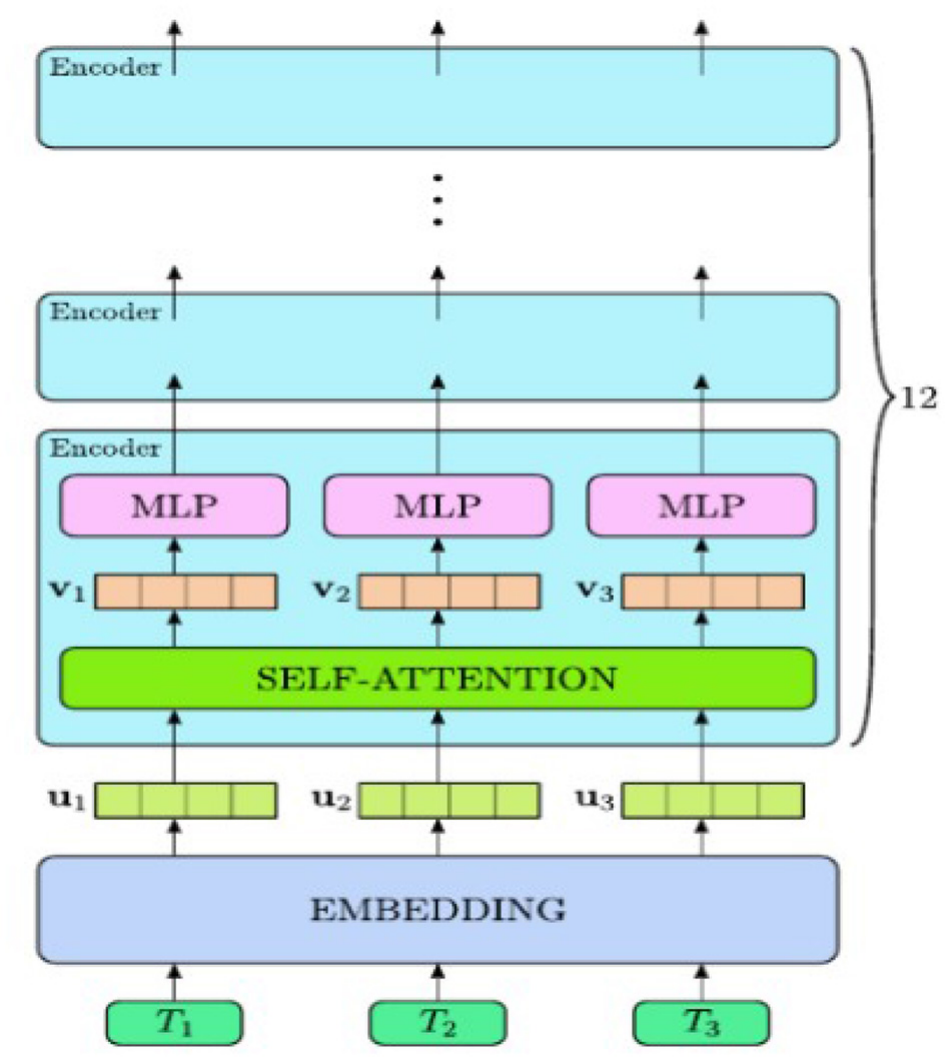
BERT model (Schönfelder and König, 2021).

Schönfelder and König (2021) proposed a Natural Language Processing (NLP) method to enable automatic extraction of design constraints from German construction codes using a fine-tuned BERT variant model for Named Entity Recognition (NER). Evaluation of 2,500 of the annotated sentences revealed the fine-tuned transformer model achieved over 95 percent precision and recall for all twelve specialized classes. The domain adapted transformer method also handled unstructured legal text efficiently with little effort for annotating the extracted information, ultimately streamlining the extraction of data related to regulations and facilitating the feeding of construction-related information into machines. Figure 2 shows the BERT model architecture.

Mukherjee et al. (2023) exhibited BERT and affiliated transformer models to create an intelligent data management system for the oil and gas industry that utilized structured and unstructured data pipelines performing Named Entity Recognition (NER), relation extraction, and operational classification designed to assist in decision-making and continuous monitoring of drilling and production activities. By automating such tasks, Natural Language Processing (NLP) provides for faster data retrieval, greater collaboration among stakeholders, and enhanced transparency in industrial processes.

Bharadiya (2023) next demonstrated an example of Natural Language Processing (NLP) powered Business Intelligence (BI) tools that provide sentiment analysis, text mining and entity recognition and convert large volumes of textual data into producing business insights that can stimulate data-driven strategies. Such business intelligence (BI) tools also expose hidden trends and patterns in unrelated unstructured data sources (e.g., social media, customer feedback) all through the incorporation of natural language interfaces that promote BI platforms to be easier and more intuitive enhancing access to data for business and non-technical users for data interactions in a conversation without the need for technical intermediates.

Dhivyashree et al. (2021) designed a BPL to handle heterogeneous and chaotic social media data. The unstructured data, which includes text, numbers, hashtags and symbols is preprocessed and structured using foundational NLP tools. Then, an application of the Markov decision process (MDP) is used to translate these natural language inputs into SQL queries to make data retrieval simple and convenient.

### 2.3 Business Process Modeling and automation

NLP is essential for Business Process Modeling (BPM) and Business Process Redesign (BPR), assisting organizations with workflow automation and improving operational efficiency.

Mustansir et al. (2022) proposed an NLP-based algorithm to consolidate user feedback, implementing a deep-learning approach for recommendation segmentation and annotation. Likewise, Godbole et al. (2021) studied automated BPM systems use NLP to capture the textual data and convert it to usable insights, such as sentiment analysis and data structuring.

Sintoris and Vergidis (2017) described an approach that lets one get BPM models from organizational documents through an automatic analysis of sentence syntactic and grammatical structure and, thus, draw BPMN (Business Process Modeling Notation) diagrams.

According to Ivanchikj et al. (2020), BPMN Sketch Miner fuses constrained natural language note-taking with process mining to formulate BPMN diagrams in real time. Elmasseer et al. (2023) further developed the process using Probabilistic Latent Semantic Analysis (PLSA) to automatically develop accurate BPMN diagrams from textual descriptions and reduced human error.

Bordignon et al. (2018) identify six main NPL techniques in business process management: text structuring, tokenization, lemmatization, information extraction, measure of similarity, and lexiconizing with databases such as WordNet and VerbNet. In combination, these techniques allow for accurate and automated development process models.

Han et al. (2019) used contextual pre-processing (CK-PP) along with optical character recognition (OCR) and Faster R-CNN to created a part-of-speech (POS) tagging tool for Business Process Documents with potential improvements in accuracy. The tags created from the developed programs had advantages, compared to a generic POS tagger, suggesting potential improvement in domain specific NLP models.

Ferrari et al. (2019) created QuOD, an NLP tool designed to automate the evaluation of documents and for grammatical correction (e.g., clarity, subscriber uncertainty, and simplification) of business process documents. The application reduces ambiguity, improves language simplification, and ensures clarity across stakeholders in the process. Using QuOD there exists improvements in communication and efficiency.

Through this paper, Elmanaseer and Rand (2023), propose an approach for automatically generating Business Process Modeling Notation (BPMN) diagrams from natural language process descriptions using Natural Language Processing (NLP). BPMN is a notation for expressing business processes in a standardized language, and this allows organizations to understand, analyse, and improve workflows. Generating a BPMN diagram, however, proves to be a cumbersome as well as error-prone process. By using NLP, particularly Probabilistic Latent Semantic Analysis (PLSA) to treat ambiguity in language, the proposed method translates textual requirements into structured BPMN elements. This helps not only in creating BPMN diagrams but also in accuracy and efficiency regarding business process representation, which will help the organizations in their estimation of resources, system comparison, and process optimization. Therefore, it aims to correct former limitations of the research and introduce a new direction in the process modeling approach using a novel NLP-based approach for driving the further advancement of process modeling toward wide applications in industries.

The paper by Taymouri et al. (2021) analyses how heterogeneity within the organizations often occurred within their business processes, in which variations in performance may be due to contextual, human, or business factors. These are called process variants, and this is the difference in the performance that happens for quote-to-cash and for insurance claims handling processes. Analysts and managers would need to know such kinds of variation in order to standardize or even enhance the processes. A comprehensive review of the literature on methods of process variant analysis based on inputs, outputs, analysis purposes, algorithms, and extra-functional characteristics distinguishes one from another. The most fundamental contributions are in the deviation found between process models and their executions, the encoding of behavioral differences in natural languages, and the classification into two big families of algorithms based upon: process mining and FCA-based ones. The results of a process variant analysis tend to focus on explanations: how the different variants can be explained by rules or causal relationships and assisting in decision-making about the process improvement.

### 2.4 Integration with IoT, AI, and ethical governance

A study highlighting how the combination of NLP and AI, integrated with IoT technologies, has an impact on business communications and how it transformed the customer interactions in the digital era was done by Mah et al. (2022). With everything including transition business processes moving online, NLP and AI now play a crucial role in understanding and meeting the needs of the customers through text, voice, and smart technologies. The research is more focused on how companies use these technologies to capture customer insights and influence engagement. From the results we can see that there is moderate effectiveness (12 out of 15) in how IoTs impact customer behavior in Industry 4.0. From the findings of Pascal, we can confirm that NLP and AI are essential for enterprise management to enhance customer satisfaction and in achieving business goals. The study reveals that rather than investing in costly advertisements, companies have the opportunity to leverage NLP and AI to analyze customer feedback from social media and other digital channels, which allows for more targeted efficient marketing strategies.

This research by Rathore (2023) focuses on the impact of the integration of artificial intelligence and the metaverse in the fashion industry, especially marketing and business operations. The metaverse is a space that has possibilities for improved virtual interaction, secure online transactions, and revenue generation. In the fashion world, AI facilitates prediction of trends, customization of customer experiences, and the optimization of inventory and logistics with data-driven insights. Applications such as virtual fitting rooms and AI-based recommendation systems improve customer satisfaction through personalization of the shopping experience. The study demonstrates AI-based strategies like design, customer segmentation, and optimization of real-time pricing for more efficient operations and competition in the industry. By creating a need for sector-wide reforms and skill-building activities to fully exploit AI benefits, the study appeals for further study of its financial benefits to the garment industry.

This paper explores the prospect of ethically governed AI to enhance finance and marketing systems in business organizations. As it is now recognized that AI enhances efficiency and productivity, the ethical implications that arise from decisions made make it a valid reason to develop an ethical framework that incorporates AI with principles of transparency, accountability, and fairness. The proposed system by Adarsh et al. (2023) will handle a huge amount of data in aiding informed decisions and is going to be based on case studies of business leaders and AI experts, and data gathered through questionnaires and interviews. The research identifies advantages and ethical issues with the use of AI in business processes, thereby generating recommendations for responsible use of AI, and get better insight into norms in business decision-making on ethics. This creates a new approach that could lead to gaining a comparative advantage in the way businesses work while having ethical integrity.

In the paper by Mishra et al. (2023), discussed about PROMPTAID which is a visual analytics system designed to allow non-experts to efficiently design, refine, and test prompts for Large Language Models in the absence of difficulties associated with creating optimal prompts out of the linguistic structure and differences of context. It presents the users with partially automated strategies like keyword suggestions, paraphrasing, and in-context examples that are inter-actively available, empowering users to explore and modify prompt templates interactively with lesser cognitive overhead. The system was developed through iterative prototyping with NLP experts, supports masked and generative language models, and is designed to make the user's work more efficient beyond that of current prompting interfaces while still allowing for better task performance without deep domain knowledge.

The applicability of NLP in business is primarily for improving decision-making, customer engagement, and process automation. Key NLP models, such as BERT, GPT, and LSTM, are at the forefront of transforming sentiment analysis, CRM, and business intelligence. NLP holds great potential as its integration with AI and IoT will ultimately lead to intelligent, adaptive systems. Additionally, increasing attention on ethical AI communicates the importance of transparent and responsible use of AI. In conclusion, NLP continues to provide organizations with a more efficient operation and intelligent consumer-based responses, while maintaining relevance in a data-driven economy.

## 3 NLP in healthcare

### 3.1 Knowledge extraction and smart healthcare

Basyal et al. (2020) emphasized how NLP allows knowledge extraction from unstructured healthcare sources such as prescription notes and clinical notes to facilitate informed decisions and the potential for improved outcomes. The article highlighted the need to continue to refine and improve NLP models for understanding complicated text by increasing accuracy and avoiding pitfalls, including an incomplete understanding of critical terms. Table 2 shows the micro application of NLP in Healthcare.

**Table 2:** Micro-Applications of NLP in Healthcare.

**Domain**	**Micro-applications**
Healthcare	Knowledge extraction
	Smart healthcare
	Electronic Health Records (EHRs)
	Social Determinants of Health (SDOH)
	Radiology and imaging
	Predictive modeling
	Mental health care
	Clinical Search Functionality
	Cybersecurity

Zhou et al. (2022) examined the role of NLP in smart healthcare settings and solutions across domains such as clinical practice, strategic development, management, and public health, including its application in public health during the COVID-19 pandemic. The study recommended studying and better integrating multiple NLP algorithms and leveraging the influence of the various data types on the processing of, and the implementation of, NLP.

Recent developments in transformer models and transfer learning have enhanced NLP's capacity to handle large datasets, though efficient training remains a challenge. Zhang et al. (2022) notes that successful deployment of ML in healthcare relies on robust data collection, organization, and infrastructure, all of which are essential for overcoming current NLP limitations and fully realizing ML's potential in clinical applications.

Alowais et al. (2023) described the transition to artificial intelligence-based systems dependent on large language models, emphasizing their importance within diagnosis, clinical decision-making assistance, and medication considerations. The authors also emphasized making certain that ethical issues, privacy concerns, and data security challenges, were considered when developing these systems so that artificial intelligence can be implemented in a safe manner within healthcare.

Subrahmanya et al. (2022) detail ways that data science, including natural language processing, may bridge the healthcare-based context with predictive analytics and personalized treatment, despite concerns regarding issues related to data heterogeneity. Predictive analytics and personalized treatment can contribute to patient-centered healthcare and improve efficiency within healthcare as a whole.

### 3.2 Electronic Health Records (EHR) and predictive modeling

Hossain et al. (2023) conducted a review of 127 studies that involved Natural Language Processing, Machine Learning, and Deep Learning in Electronic Health Records (EHRs). They identified issues relating to limited annotated data, poor model quality, and data imbalance as continuing challenges. They then proposed that future research be directed mainly to advancing classification and prediction tasks, including ICD-9 coding and clinical prediction.

Reeves et al. (2021) wrote about their work developing an NLP tool called *Moonstone* which was designed to extract Social Determinants of Health (SDoH) indicators from EHRs with precision and recall scores of 0.83 and 0.74, respectively. The tool's flexibility in different clinical contexts may support improved implementation of SDoH into healthcare data systems.

Tayefi et al. (2021) noted that Electronic Health Records (EHRs) have structured and unstructured elements, both of which contribute to health care knowledge. Connectionist models, such as NLP, ML, and radiomics, have emerged as commonly used technologies for processing structured and unstructured data, but the challenges of privacy, data quality, and access to data still exist. The authors also advocated for making unstructured data analysis capabilities easier by implementing deidentification, pseudonymization, and synthesizing anonymized data for secure and valid use.

Yang et al. (2022b) developed **GatorTron**, a clinical language model on a large scale with a training set containing over 90 billion words that included deidentified medical terminology. On five NLP tasks, their GatorTron clinical language model performed better than similar models like BioBERT and ClinicalBERT. GatorTron achieved improvements in accuracy in the medical domains of natural language inference and medical question answering, while supporting scaling of both the model parameters and the data delivery capabilities in high-quality clinical AI systems.

Arnaud et al. (2021) used NLP for the early prediction of medical specialty needed for patients at the time of admission to the hospital, using data from over 260K records of emergency department visits. They developed a hybrid model that included structured data and CNN-based textual analyses that had reported high accuracy overall. These two conclusions illustrate that NLP has potential to assist hospitals in clinical decision-making and improve operational efficiency.

### 3.3 Radiology, imaging, and clinical applications

**Figure 3:**
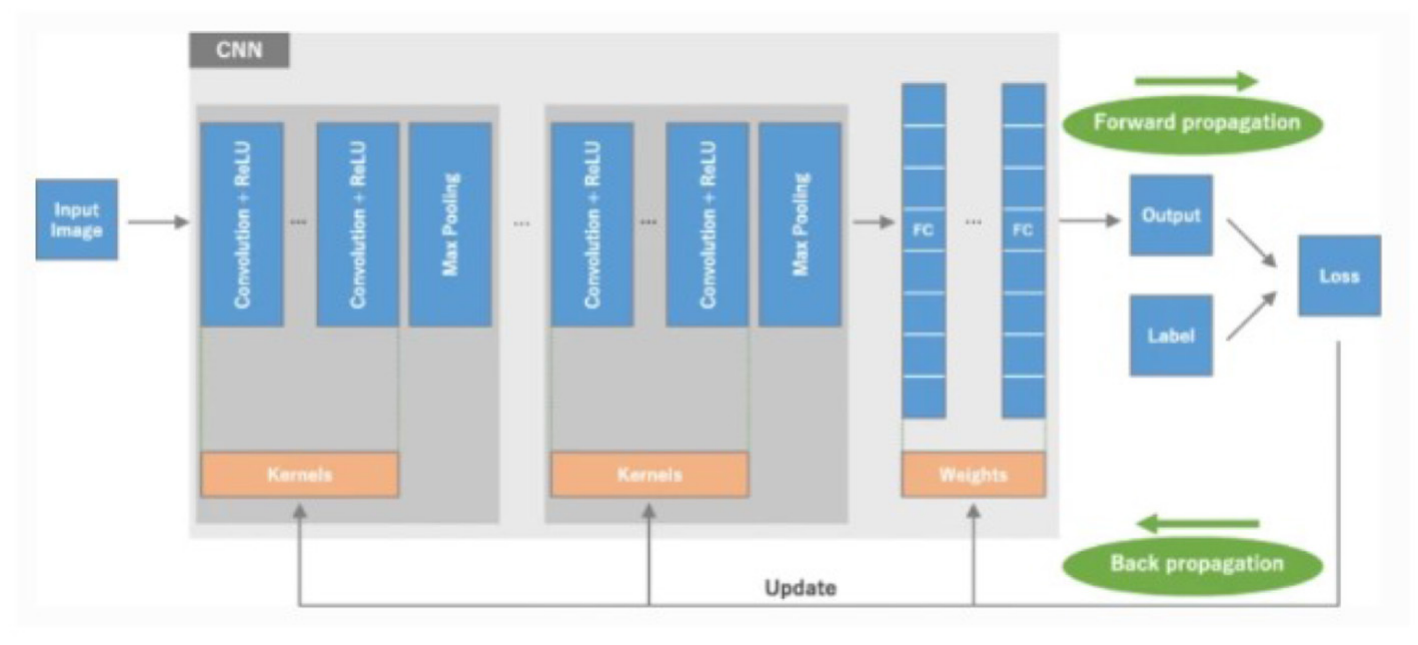
Architecture of CNN (Yamashita et al., 2018).

Convolutional Neural Networks, or CNNs in short, happen to be one of the highest-ranked deep learning models in all computer vision tasks and are extremely important in radiology for lesion detection, classification, segmentation of lesions, and image reconstruction. CNNs automatically learn adaptive features from images through layers consisting of convolutional, pooling, and fully connected layers. Yamashita et al. (2018) explains that unlike the traditional radiomics, which is based on handcrafted features and segmentation of professionals, CNNs do not need manual feature extraction, although they are computationally very demanding and require massive datasets. This review outlines the basics of CNN and its challenges, including limited datasets and overfitting, along with strategies to overcome them, underscoring the potential of CNN in aiding the diagnosis process of radiologists and, subsequently, patients. Figure 3 shows the working architecture of CNN.

Elbattah et al. (2021) reviewed the increasing use of *Text Analytics* in the health and medical fields for processing unstructured data to help clincians with decision-making, and to retrieve information from EHRs and predictive modeling with NLP and machine learning. A case study conducted by Tvardik et al. (2018) demonstrated the capabilities of this technology, achieving 84% accuracy for medical event detection using EHR data. Park et al. (2022) describe how natural language processing-enhanced search improves information retrieval from EHRs when compared to traditional string search. In a study using 35 evaluators, Park et al. (2022) found NLP enhanced search to produce 5.13% higher accuracy and concurrently higher satisfaction, while speed was slightly improved. The conclusion of these studies provided evidence that NLP-based search improves clinicians reliability and significantly enhances clinical efficiency & cognitive load.

Abbasi (2024) looks at the impact Artificial Intelligence (AI) and Natural Language Processing (NLP) are having on telemedicine and Remote Patient Monitoring (RPM). AI can be used to monitor health parameters in real time with the aim of detecting diseases in their early stages, whereas NLP-enabled assistants can support medical triage, preliminary diagnosis and non-clinical communication–which carries implications for reducing workload in clinicians and improving access to care in underserved geographic areas. The author underlines that data must be used securely to ensure privacy and to prevent bias for the purpose of equitable healthcare. The author advocates for innovation, and to further this aim, collaboration between healthcare and technology to maximize the benefits of artificial intelligence for patient care.

Al-Garadi et al. (2022) analyzed Natural Language Processing (NLP) in the COVID-19 pandemic, demonstrating how models such as BERT facilitated our ability to quantify health trends, track trajectories, and create evidence against health misinformation. The authors recognized that while NLP can help inform pandemic responses, they raised concerns about the limitations of the data used to train models, bias within the data, and the models' clinical relevance.

### 3.4 LLMs, mental health, and ethical governance

**Figure 4:**
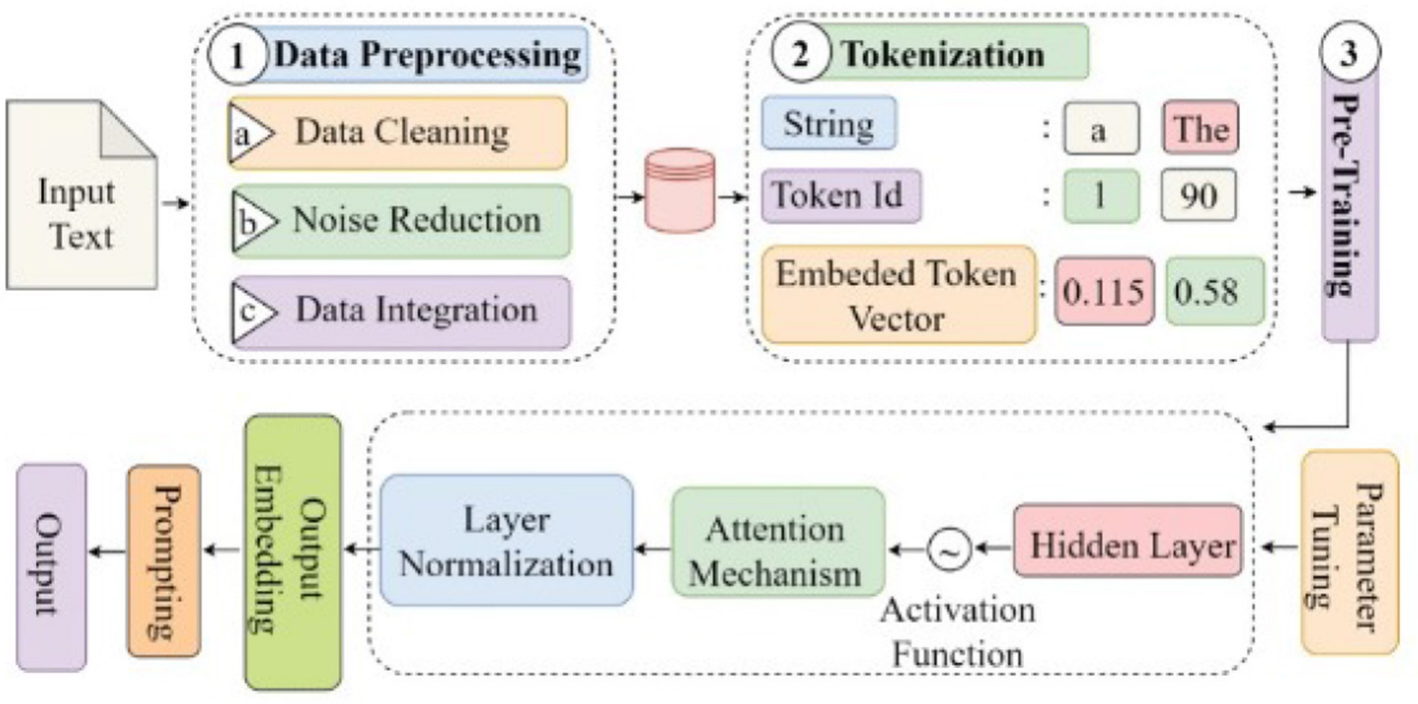
Working of LLM models (Raiaan et al., 2024).

Large Language Models have provided a revolution in NLP as such that now, even the most complex language tasks can be carried out by a machine: text generation, translation, and question answering with uncanny precision. Huge neural networks, transformer-based architectures, and enormous datasets support these models, capturing even the most intricate language patterns (Sousa, 2022). The review by Raiaan et al. (2024) encompasses the evolution of LLMs; from foundational components such as tokenization, attention mechanisms, and training techniques and newly emerging fine-tuning approaches to model bias, privacy concerns, and robust, ethical deployment practices that are new challenges coming with LLMs. Beyond revolutionizing all areas of applied science where AI applications have been deemed an essential tool, LLMs carry new challenges. The study is a comprehensive resource with insights into the evolution of LLMs and their potential to drive future innovation in each type of application field. Figure 4 shows working of LLM Models.

Nazi and Peng (2024) presented a review of the progress of **Large Language Models (LLMs)** in healthcare, citing their ability to assist and complete healthcare tasks such as entity recognition, relation extraction, and medical question answering. Despite their promise in many of these areas, biases, support for data privacy, and a need for quality training data are still issues to be dealt with in relation to LLMs.

Sinha et al. (2023) studied **ChatGPT** on 100 diagnostic pathology questions, with the overall result being approximately an 80% accuracy rate and good reasoning. Studies done by Gilson and Kung further show that ChatGPT can pass the U.S. Medical Licensing Exam, and once validated through studies and assessments, suggests the future of medical education and clinical reasoning.

LLMs are also used in systematic mental health care. Hua et al. (2024) reviewed 34 studies, and got to know the use of LLM in diagnosis, therapy, and engagement of patients, with some challenges like that of data availability, easy understanding of mental states, and evaluation methods. It is important to address these issues by using robust datasets, standardized evaluations, and interdisciplinary collaborations to realize the full potential of LLMs' in mental health care.

Similarly. Lee et al. (2021) devised the concept of “Artificial Wisdom” (AW) aims to introduce empathy and moral decision-making into AI, focusing on compassionate care aligned with societal values. Initial applications, such as robotic assistants in eldercare, illustrate progress toward integrating wise AI using NLP into healthcare. NLP converts unstructured clinical notes into structured data, enhancing diagnosis and treatment, even though challenges like privacy, ethics, and bias remain as significant barriers.

Use of NLP also maintains precautions against the risks of bias and discrimination that may make health inequities worse. Leslie et al. (2021), noted that disadvantaged communities have been exponentially impacted by the pandemic due to systemic factors like racism and inequality. If NLP technologies analyse biased clinical notes, they may discriminate indefinitely when used in AI models trained on incorrect datasets from electronic health records. To handle these issues, the article supports the robust bias detection in AI development, community involvement, and careful interpretation of AI outputs, emphasizing the need for inclusive policies and addressing systemic inequities to ensure that AI contributes positively to health outcomes instead of reinforcing existing disparities.

In practical scenarios, federated learning has an impact on health care with regard to a method of tapping into diversified, broken, and fragmented data without revealing their privacy. Xu et al. (2021) explain that the ability of federated learning enables collaborating parties within the healthcare providers, insurance firms, and pharmaceutical companies to realize data-driven improvements in care. Some examples are as follows: Natural Language Processing on mobile devices, personalized retail recommendations, and financial risk assessments. It concentrates on issues related to the maintenance of data quality, bringing expertise, participation through incentive devices, and personalization in healthcare models. The open questions remain as for the refining of precision for the model, standardizing data quality, and incentives for the improvement in wearable device data. Federated learning shows much promise for making personalized healthcare and prediction models in patient care.

NLP also contributes to cybersecurity by protecting sensitive medical information through the threat analysis, user behavior, and anomaly detection. Mijwil et al. (2023) suggests use of encryption, access controls, and compliance with standards like HIPAA, NLP-driven solutions improve data security and privacy. Overall, NLP empowers a range of industries, from healthcare to cybersecurity, with the potential to drive efficiency, enhance data accessibility, and support data protection in the digital age.

The amalgamation of artificial intelligence, data science, and Natural Language Processing in healthcare is affecting care delivery, clinical diagnosis, and decision-making. A number of studies have demonstrated how electronic health records, text analytics, and predictive analytics can result in clinical insights from large amounts of structured and unstructured clinical data. Nonetheless, aspects such as data quality, privacy, and concerns over ethical use of models persist. Newer advanced large language models, such as ChatGPT, have demonstrated robust performance across competency areas such as medical reasoning, medical education, and information extraction, which indicates their rapidly growing role in clinical workflows. However, before we can safely and effectively use generative AI for clinical workflows, it remains paramount to ensure fairness in the model, security around the data, and sufficient validation.

## 4 NLP in sports

### 4.1 NLP and Machine Learning in Sports Analytics

Heaton and Prasenjit (2022) utilized both **NLP and Computer Vision** together to evaluate player influence and outcomes in the Major League Baseball (MLB) by approaching the statistical data with a paradigm of analyzing sequences of events rather than just summary statistics. Their model produced **player form embeddings** which allowed the model to consider the players short- and long-term performance in the analysis of predicting game outcomes.

Weissbock and Inkpen (2014) were also able to previously use **NLP and sentiment analysis** from sentiment analysis of pre-game NHL reports to predict outcomes of games. As a result of combining the sentiment data with more traditional statistical features, it able to create models that were successful in predicting outcomes of games at a rate of **60.25%**. The implications highlight the value of NLP when combined with traditional methods of analytics.

Xia et al. (2024) conducted a review on **NLP multimodal models and sports analytics** and were able to categorize datasets into **language-based, multimodal, and convertible type datasets** for fan engagement, tactical analysis, and diagnostics. They raised the importance of the significance of findings using large-language models or LLMs, similar to GPT and Llama2, for tasks such as summarization and predicting hate speech, while acknowledging there are still challenges in building real-time datasets with diverse high-quality data and modes in the future of sport.

### 4.2 NLP adoption, education, and evaluation in sports

Through his paper, Wanless et al. (2022) uses the theory of diffusion of innovations to examine the adoption of Natural Language Processing (NLP) in professional sports. NLP involves training algorithms for text pattern recognition. It is very important nowadays due to the growing volume of text data in sports. Using a combination of the Bass model, literature review, and qualitative analysis, the researcher explores the mechanisms, timings and influences behind the adoption of any NLP technique. The study indicated a huge jump in NLP and a need for a proper framework for further academic and practical exploration of NLP in the sports industry. Table 3 shows the micro application of NLP in sports.

**Table 3:** Micro-applications of NLP in sports.

**Domain**	**Micro-applications**
Sports	Performance analytics
	Fan engagement
	Biomechanics analysis
	Teaching evaluation in sports education
	Video analysis in sports
	Game prediction

A unique implementation of NLP in teaching is Student Evaluation of Teaching (SET) used to assess and enhance the quality of teaching, especially in sports education in China. SET studies are widely used and are focused on structured data(like rating scales), rather than unstructured data or feedback. Li et al. 2022 analyzed the descriptive SET data from several universities in order to identify key traits for evaluating sports teaching done through Natural Language Processing (NLP) and Latent Dirichlet Allocation (LDA). After examination of unstructured comments, the study highlights how NLP provides valuable insights, which support improvements in sports education through a multidimensional view of SET data (Bunker and Susnjak, 2022).

In their 2022 study, (Yang et al. 2022a),(b) presented **ChatMatch (CM)** as a framework for the interactive assessment of chatbots based on their conversational performance. The ChatMatch framework differs from more traditional frameworks that are either static in their evaluations or human judged by allowing **multiple bots to compete against other bots in a tournament structure**, while using common measure metrics. The framework accommodates different acceptable responses systematically, closely aligns with human judgement, and provides a cost and scale efficient alternative to various costly manual evaluations.

### 4.3 NLP, vision, and deep learning models in sports performance

Sports presents a significant challenge for NLP in understanding it due to its complexity and dynamic nature. A paper by Yang et al. (2024) evaluates mainstream large language models (LLMs) and video language models (VLMs) for various sports related tasks. These tasks can be basic rule queries to complex context-specific reasoning. The studies show two types of approaches—unimodal and multimodal, including zero-shot and few-shot learning techniques. From the results we came to know that LLMs can handle basic sports understanding well, but they are not so good with advanced scenarios like multi-hop question answering. The best results in complex tasks involved chain-of-thought prompting. The research is able to identify critical challenges NLP face in sports understandings and proposed a new benchmark based on existing datasets. Multi-modal fine-tuning of VLMs will be considered as future scope, enhancing interpretability in sports officiating systems.

**Figure 5:**
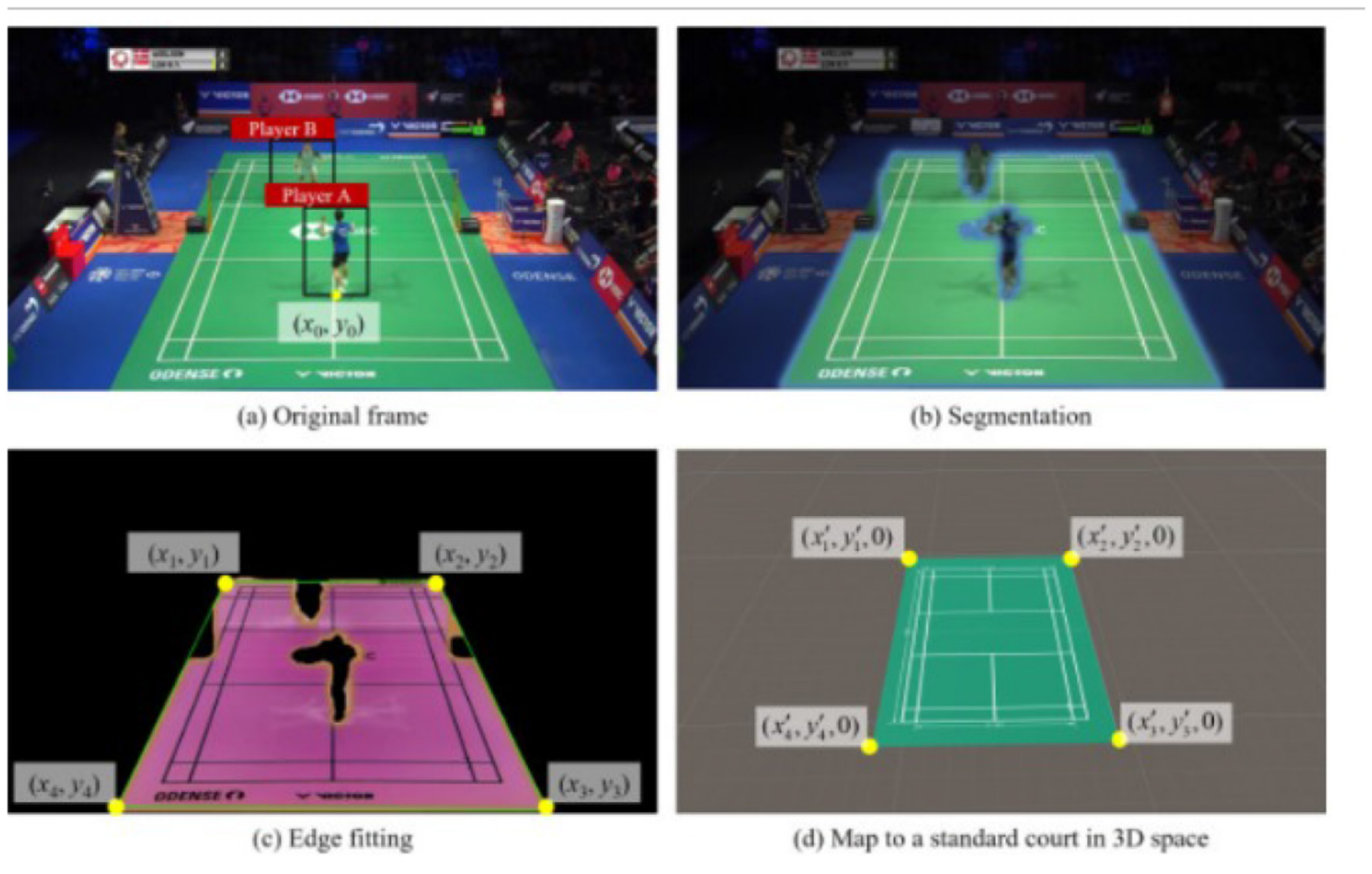
Extracting space information from images (Zhang et al., 2025). **(a)** Original frame. **(b)** Segmentation. **(c)** Edge fitting. **(d)** Map to a standard court in 3D space.

**Figure 6:**
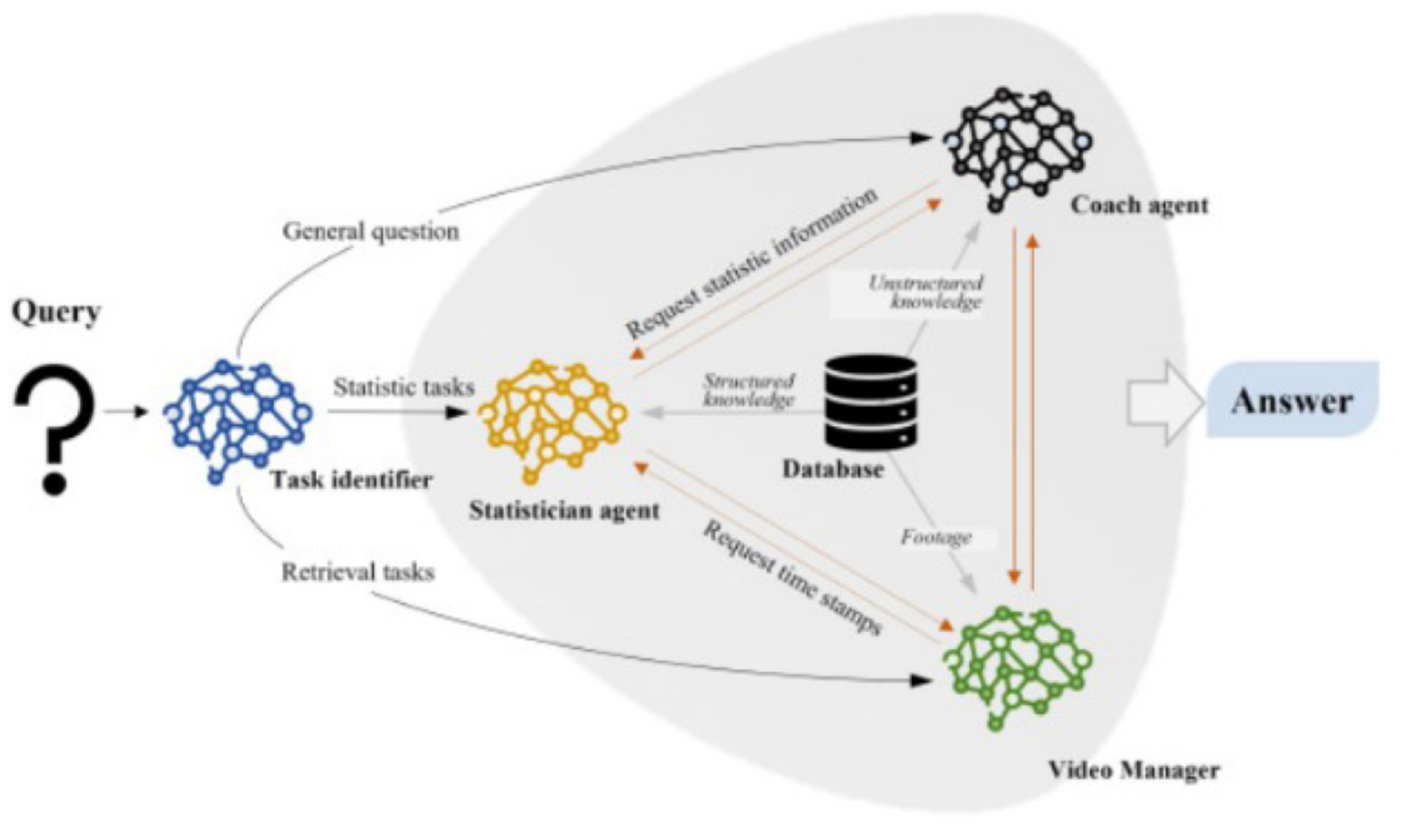
Extracting space information from images (Zhang et al., 2025).

NLP based Chatbots for sports analysis are also quite famous. ChatMatch developed by (Zhang et al. 2025), is a video analysis framework used for racket sports and is powered by Artificial Intelligence, that combines vision and language models, enhancing video comprehension. It uses deep learning for extraction of detailed player actions, locations, and gestures, while LLM-based agents collaborate their work to answer user inquiries independently. Validated on badminton, the system was able to achieve high accuracy in identifying player features and responding to professional questions. The study reviews existing and possible limitations in near- and far-field analysis, where ChatMatch helps in providing a unique solution for comprehensive video understanding, supporting not only elite, but also grassroots sports analysis. Figures 5, 6 map to a standard court in 3D space and extracting space information.

**Figure 7:**
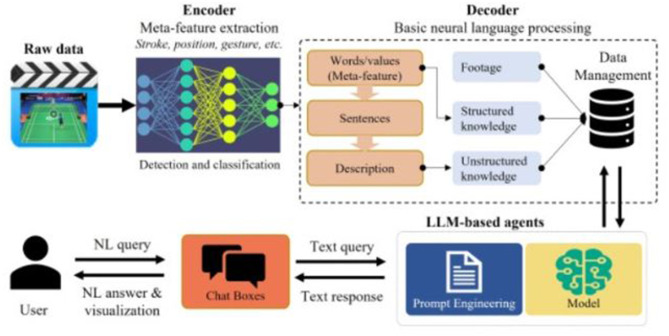
Extracting Space information from images. Zhang et al. (2025).

Another NLP framework widely used in Sports is TCN. (Lea et al. 2017) introduced the Temporal Convolutional Networks, as an efficient alternative for RNN-based models in action segmentation, going to capture long-range temporal dependencies and action compositions and resolve the challenges faced in segmenting fine-grained human actions. Two variants of the TCNs are presented: the Encoder-Decoder TCN makes use of pooling and upsampling for long-range temporal patterns, and the Dilated TCN takes advantage of the dilated convolutions of the WaveNet model. These models outperform traditional methods, like Bidirectional LSTM, since they are faster to train and contain fewer segmentation errors. What can be concluded from these results is that TCNs deliver state-of-the-art performance when tested on several challenging datasets and are, therefore, very promising as an alternative to recurrent neural networks. Figure 7 show the process of encoder and decoder used in the agents.

### 4.4 Data mining and biomechanics in sports science

Using data mining and Natural Language Processing and based on the analysis of more than 1,400 papers, Zhang and Fan (2024) examined sports biomechanics research in China (1980–2022) with the aim of identifying major trends and interdisciplinary integration. Focus areas included, on the mesoscopic level, technical analysis, athletic training, and prevention of injuries in strong connection with physical education and the acquisition of motor skills. There are increasingly strong links with biomedical engineering and computer applications underlining a bright and dynamically changing future for sports biomechanics as a multidisciplinary field.

The main application of TCNs is sports training and result prediction. For example Xi et al. (2024) presents a dual-channel architecture combining a Spatiotemporal Transformer and Temporal Convolutional Network (TCN) for enhanced 3D human pose estimation in sports within IoT environments. The model effectively handles fast, complex movements by integrating global and local spatiotemporal features. It is tested on datasets such as Human3.6M and MPI-INF-3DHP, which helps it outperform existing methods in terms of accuracy and real-time efficiency. As it is computationally demanding and less adaptable to diverse environments, its future scope is to optimize the performance and broaden applications to fields like rehabilitation and smart sports.

The present section offers a review of some of the recent developments that combine Natural Language Processing (NLP) with sports analytics, biomechanics, and education (Li et al., 2023). The literature highlights NLP used with machine learning and computer vision to assess player performance, predict outcomes of games, and to assess tactics within games. Research has also highlighted the use of NLP in sports biomechanics to track trends and predict injuries. In addition, NLP assists in sports education with assessing feedback and improvements in instruction. Additionally, models such as ChatMatch show practical ways of evaluating conversational systems within sports applications. Overall, NLP offers a way to build data-driven, interactive, and interdisciplinary approaches to improve research and practice in sports.

## 5 NLP in education

### 5.1 NLP for learning assessment and student performance

Crossley et al. (2017) investigates the relationship between linguistic feature and math performance in collaboration problem-solving among second language (L2) English speakers using an online math tutoring system. For an analysis of the transcribed speech, this study employed NLP tools in order to review text cohesion, lexical sophistication, and sentiment as predictors of mathematics performance. Results: As shown in the Table 4, the results proved that non-linguistic factors like gender, age, grade, and content focus were poor predictors, while linguistic features explained a 30% variance in match scores. That shows how with language proficiency greatly influencing match performance, the direction of learning outcomes encourages or requires cohesion and lexical sophistication.

**Table 4:** Micro-applications of NLP in education.

**Domain**	**Micro-applications**
Education	Automated grading and assessment
	Personalized learning
	Student Evaluation of Teaching (SET)
	Text summarization for accessibility
	Virtual learning assistance
	Content customization
	Automated question generation

This work differs in the challenge of student summary evaluation by developing an NLP-based automated summarization evaluation(ASE) model based on openly available, text-based NLP features so that this model may be generalized and reproduced. Employing over 1,000 summaries from different prompts allowed us to achieve a proportions that were classed as either high or low quality greater than 80%. Unlike previous methods which rely heavily on expert annotation or proprietary resources, Crossley et al. (2019) focuses on linguistic features of content integration, coherence, and language use, making it suitable for dynamic classroom environments. The ASE model should enhance online tutoring systems by providing automated formative feedback to allow students to summarize more effectively.

Natural Language Processing, in reality, has transformed education because it has armed the learning environment with tools for analyzing and interpreting large amounts of qualitative data in a more efficient way. NLP can help automate the process of assessment, recommendations, and tailored feedback depending on students' needs. For instance, NLP can help point out patterns in student responses and make content analysis easier, thereby enabling educators to discover insights that improve their teaching strategies. For instance, Zhai et al. (2021) give thorough review of accessibility of NLP-based systems, like text-to-diagram converters for visually challenged students, opens the doors of greater access and inclusion. Educational tools may respond dynamically to the inputs made by the learner by using advanced NLP models.

### 5.2 NLP for educational content and accessibility

Lucy et al. (2020) analyzed 15 most widely used U.S. history textbooks in Texas classrooms between 2015 and 2017 with respect to the treatment of marginalized historical groups using NLP tools, such as lexicons, word embeddings, and topic modeling, yielding the following results: Latinx people were named very seldom, and famous figures from the past were predominantly White males. New NLP methods stress the promise they offer in interpreting textbook content; it give one a new perspective regarding educational discourse while recognizing familiar methods.

In his paper, Crowston et al. (2012) explores how NLP can help with qualitative research by automating aspects of content analysis and lessening the amount of manual effort. A case study demonstrates the effectiveness of applying NLP rules to code text, achieving high accuracy for several codes. Present-day capabilities of NLP now reduce the volume of text that needs to be examined by hand by an order of magnitude, accelerating the coding process quite significantly. This study shows that bringing advanced NLP techniques may brighten the process of qualitative data analysis, which is an early step in introducing advanced computational tools into social research.

A cataloging tool that uses Natural Language Processing (NLP) techniques to assign educational standards metadata to digital library resources, addressing some of the complexity that is brought about by variability at the national, state, and local levels was introduced much before. Devaul et al. (2011) determines whether the tool suggests standards matching human assignments, ranks appropriate standards correctly, and increases relevance with inclusion of metadata in queries. The tool should focus on improving the cataloging process, which may make workflows easier and more effectively support teaching, learning, and accountability in linking standards to digital resources.

### 5.3 AI, LLMs, and NLP in modern education

AI, with specific emphasis on NLP and large language models (LLMs) like GPT-4 and BARD, in education and research. Alqahtani et al. (2023) explored how AI enables one to make strides in areas of personalized learning, automatized assessments, and offering support for mental health needs of students, thereby maximally increasing student engagement and results. In research, AI aids in drafting scientific text, analyzing data, and providing discoveries, especially in areas like bioinformatics and drug discovery. Responsible AI integration would mean accounting for issues such as ethics, data privacy, and algorithmic bias. On the whole, the paper advocates for a balanced use of AI in education and research, thereby providing adequate training and resources for the stakeholders on using it effectively.

AI and EdTech are shifting the paradigms of learning, transforming the conventional “one size fits all” style toward more personalized and student-centric learning. Bhutoria (2022) take examples of China, India, and the USA to highlight how AI facilitates personalized learning for each individual based on needs, habits, and capabilities of each learner. The study through the application of NLP and topic modeling identifies key trends where AI enhances learning in diagnosing and predicting challenges, content customization, and student engagement through VR and other technologies. This need for equitable and reliable integration of AI in education persists against the problems of data privacy, accessibility of resources, and cost.

Latin America uses many applications of NLP in its higher education like ranging from chatbots to the content analysis and sentiment analysis application, among others. The NLP automates processing of vast volumes of text data, which happens to be very beneficial for an area, for instance the students' evaluation process or any documentation analyses or evaluating teacher review processes. Salas-Pilco and Yang (2022) notes how it improves access to education and enhances the personalized learning experience by providing real-time assistance and feedback. NLP applications are slower to adopt than other fields but contribute significantly to student performance, teaching support, and administrative efficiencies. However, challenges remain around ethics and data privacy, which indicates the need for clear data protection protocols in educational applications.

### 5.4 NLP and AI in educational systems and research tools

The present study evaluates NLP applications in using the MedLEE system to recognize 45 types of events defined by the New York Patient Occurrence Reporting and Tracking System, NYPORTS, against adverse events in electronic discharge summaries. The performance of the system is assessed by Melton and Hripcsak (2005) against manual reviews on a sample of 1,000 discharge summaries and a larger set of 57,452 cases over two years. The system had high specificity (0.985) but only moderate sensitivity (0.28), correctly identifying 704 of 1,590 apparent events and had much higher sensitivity than traditional reporting methods. Although the sensitivity was fair, a high specificity of the system to detect a wide range of complex events suggests a good possibility for enhancing detection and prevention of adverse events in healthcare with minimal clinician workload and potential real-time clinical feedback.

Children can also utilize nlp to summarize youtube videos. By summarizing videos based on audio content, the system allows quicker browsing and retrieval, which enables users to select relevant videos much more easily. Nagaraj et al. (2023) developed a method involving generation of transcripts via speech recognition, scoring of sentences within these transcripts, and selection of the highest-scoring sentences. Similar video segments are then extracted and merged to create a new concise summary of video. This approach is used to enhance the efficiency of storage and improve accessibility of users to vast video libraries.

The approach proposed to be followed focuses on video description generation and timestamping. The idea of Emad et al. (2021) is that it will allow the system to automate all these processes such that, with frames, emotions, and speech summarization, the system minimizes time devoted toward searching relevant videos to ensure a user watches only portions of a video desired. Some other crucial methods of producing abstractive summaries include keyword extraction, video frame summarization, emotion analysis, and audio transcription. In the experiments, the processing was based on natural language using tokenization, sentence segmentation, lemmatization, and abstractive summarization to fuse the inputs into a meaningful summary. The outcomes of the experiment were that 87% felt that the text generated reflected the content in the video hence enhancing video search efficiency.

This study by Moon et al. (2021) proposes an advanced framework to detect and reject unnecessary or unethical video content through NLP and machine learning techniques. First, it converts the video data to text by extracting audio. Then it uses Naive Bayes and logistic regression classifiers in order to identify harmful content-such as sexual, political, criminal, or terrorist material. Through text chunking, it can use the NLP component to process unstructured texts and derive features from noun and verb groups for analyses. The proposed model is effective in video genre classification with high accuracy, while it shows its capability in determining inappropriate content for filtering. In summary, this would address the challenges brought by unethical and malicious video uploads and create a much safer online video environment.

This study applies text mining and Natural Language Processing techniques for analyzing construction accident reports to classify accident causes and identify hazardous objects, which are useful for enhancing workplace safety. According to Fan Zhang et al. (2019), five baseline classifiers are SVM, LR, KNN, DT, and Naive Bayes. An optimized ensemble model was used for the classification. The ensemble produced the best result in terms of average weighted F1 score. For extraction of common dangerous objects, a rule-based approach for chunking is proposed. The significant preprocessing steps include text normalization, stop words removal, and feature extraction. Findings explain the efficacy of automation and identify further improvements in the form of data balancing, bigram/trigram use, more advanced neural networks like LSTM, and domain-specific tools to increase levels of accuracy. The methods focus on the improvement of safety management systems and reducing construction site risks in the long term.

Progress in NLP, especially in pre-trained models like BERT and GPT, has led to transferring knowledge and adaptive, real-time educational tools that customize learning experiences. Yang et al. (2021) note how AI-driven “precision education” can identify at-risk students, support timely interventions, and create smart assessment systems that automatically generate test questions, grade answers, and provide feedback through text summarization and deep learning. The paper does, however, raise several ethical challenges, such as the biases in NLP data that might perpetuate social inequalities. Thus, these authors propose a human-centered approach to AI: designing AI with human values and understanding, which will lead to better educational outcomes and retain ethical integrity. It explores an interdisciplinary dialogue in ensuring that AI applications in education work effectively and socially responsibly.

MathBERT is a domain adaptation of BERT pre-trained over a large corpus of mathematics content, from pre-kindergarten to graduate-level material, which includes textbooks and research abstracts to address the problems within NLP over mathematics. The model by Shen et al. (2021) introduces a special vocabulary, “mathVocab,” so that it can better handle equations and mathematical symbols with much better results than BASE BERT and other methods on knowledge component prediction, auto-grading open-ended Q&A, and knowledge tracing. MathBERT outperformed BASE BERT by 2–8% and is set to be used within learning environments like ASSISTments and K12.com. Model and resources are public and, in the future, will be explored in informal math content applications for tasks like conversational tutoring.

SCIBERT is a specific variant of the general-purpose BERT model, trained on an enormous scientific corpus of scientific publications. It tries to avoid shortages of labeled data in scientific domains by using unsupervised pre training to achieve state-of-the-art performance for tasks such as named entity recognition, text classification, relation classification, and dependency parsing. SCIBERT uses both the original BERT vocabulary as well as scientific-specific vocabulary and shows substantial improvements over BERT-Base and competitive performance against BIOBERT on biomedical datasets. Future directions for Beltagy et al. (2019) in expanding SCIBERT to larger models as well as optimizing utility across different scientific domains are mentioned.

Similarly, In his work, Xu and Zhu (2023), applies the BERT model from the Natural Language Processing domain to optimize the smart teaching in higher education for computer classes by developing an intelligent test database based on the process of a bidirectional encoding, extracting semantic features from the BERT model so that there could be precise textual analysis of knowledge points. Such an approach utilized the kind of insight offered by NLP with the five-stage teaching method developed into improving student engagement, problem-solving ability, and practice skills. This paper thus documents the recent empirical performance of BERT in capturing a more accurate semantic meaning compared to conventional methods of database development, underlining this pivot toward innovative teaching methodologies informed by data in progressing educational reforms.

Koroteev (2021) reviewed BERT as a paradigm-shifting deep model in Natural Language Processing that has achieved super competence in such applications as text classification, annotation, and translation. BERT architecture, as a two-way transformer and masked language model, allows it to win context-aware text representation. BERT is more powerful than previous models, namely, word2vec and GloVe, as it can be used after pretraining on big unlabeled datasets and fine-tuning for a specific task. This paper describes great flexibility in utilizing pre-trained models for usage, multitask learning, and new results in the evaluation of generated texts by BERT, marking this as a cornerstone in modern NLP research and applications. Key benchmarks such as GLUE, SQuAD, and SWAG show remarkably consistent accuracy levels that surpass human capabilities.

Malinka et al. (2023) point out that NLP can serve as a change agent in education, enhancing student learning and teacher efficiency. Models, like GPT models, that can learn from and analyze even complex prompts and respond appropriately are responsible for the current enhancement of educational and learning processes with NLP. Data testing the use of NLP has shown to enhance the ability to solve math problems and even classify student questions by cognitive level learner. While NLP is better than tools of years gone by, there are still issues with competency in understanding user context, inconsistency, and a lack of common sense. Tools, like Chat GPT, can generate textual information and evaluation quickly but have raised concerns related to academic integrity and the validity of assessment. The researchers recommend ethical adaptation strategies to try to gain the benefits of NLP while simultaneously working to reduce its malefactors.

ChatGPT, created by OpenAI, is a popular Natural Language Processing (NLP) platform built off some of the functionalities behind a deep learning and Reinforcement Learning from Human Feedback (RLHF) model called the Generative Pre-trained Transformer (GPT). In education, ChatGPT can assist with personalized learning, grading, and generating feedback for students, which allows teachers to prioritize the most important aspects of instruction. Additionally, it can foster student critical thinking by having them consider the questions they pose to ChatGPT, as well as examine the responses to those questions. The implementation of ChatGPT may be beneficial in many fields outside of education, as noted in the work by Javaid et al. (2023) where references to customer service, content generation, and community learning is noted as primary areas where the tool can be implemented.

In a similar vein, Lund et al. (2023) provide valuable insight into the growing role the tool is playing in academia and research, as they discuss the utility of AI tools to help draft an essay and generate citations to make it easy for authors to write on topics. However, they recommend caution to avoid problems relating to bias, misinformation, copyright, and the Mattheiw Effect, which is an economic manner where bolder, already established researchers benefit more than others of lesser prestige. Lund et al. (2023) recommend responsible and transparent use of AI in academic contexts, while balancing the ethics of the innovation.

Kerlyl et al. (2006) discusses the integration of such conversational agents, also known as chatbots, into ITS, focusing on the concept of Open Learner Modeling (OLM), that would be revealed and negotiated with a student's knowledge model in order to enhance learning. They also highlight the recent advances in chatbot technologies-from early systems such as ELIZA to sophisticated frameworks like Lingubot-and develops their capability to analyse user input, adapt responses, and keep focused discussions. A Wizard-of-Oz experiment was run with 30 students, to test a pre-defined strategy and response-based negotiation system. The paper has been guided by the need to balance pedagogical goals with the engaging small talk involving chatbots and ITS in order to develop personalized learning experiences and reflection.

Michaud and McCoy (1999) proposed architecture for the ICICLE system's user knowledge modeling that integrates NLP with educational theories to enhance second language acquisition in deaf students for written English. Grounded in Vygotsky's Zone of Proximal Development and theories on interlanguage progression, this model catches both the learner's evolving grammar as well as those linguistic features situated within the learner's reach for optimal instruction. The system uses NLP for the analysis of systematic language errors and stages of skill acquisition–cognitive, associative, and autonomous–to evaluate user performance, to determine the order in which the acquisition takes place, and then to tailor tutorial feedback. Such granularity allows ICICLE dynamically to adapt to the needs of a learner: precise, context-aware feedback bridges theory and practice in education.

In 2006, they discussed the ICICLE project which is a Computer-Assisted Language Learning system that relies on the use of NLP for teaching written English as a second language to deaf students. What is more, Selinker's Interlanguage theory and Second Language Acquisition research are used within the system to model the learners' developing grammar correctly. The NLP technology enables ICICLE to process user-submitted texts and mark grammatical errors; thereby, it is able to generate tutorial feedback that is sensitive to improve learner understanding of language rules. It encourages extensive usage as users are required repeatedly to revise and resubmit their work so that the system can offer adaptive advice in context. Advanced NLP is utilized while ensuring accurate error diagnosis and providing appropriate feedback.

Artificial Intelligence (AI) and Natural Language Processing (NLP) are redefining Physical Education (PE) through the development of personalized learning and immersive experiences. Students can engage in interactive experiences away from traditional classrooms and field settings through Augmented and Virtual Reality (AR/VR) and Internet of Things (IoT). AI systems can help to predict performance where feedback can be instantaneous and construct lessons that are specific to the student's needs. Lee and Lee (2021) highlight the importance of teachers developing skills to implement this technology, which can provide rich and engaging learning, and, improve inclusivity.

Similarly, Goksel and Bozkurt (2019) highlight AI's evolution in education: pre-AI rule-based systems, to modern neural networks, which have permitted the development of NLP for adaptive learning, intelligent tutoring, and human-computer interaction that has the potential to enhance learning. They, also, emphasize the necessity of developing ethical and responsibly AI to enhance rather than replace the human aspect of education.

In the educational space, you can see the application of NLP across learning assessment, tailored feedback, content analysis, and intelligent tutoring, to name a few. Two examples are effective automated summarization, and adaptive models like MathBERT, which make learning more inclusive using systems like ICICLE. NLP also enables learning to be scalable and context aware. Along with large language models (LLM) such as ChatGPT and SCIBERT, NLP is making advancements in the areas of content creation, access, and engagement. At the same time, we have many challenges related to data privacy, ethical use of AI, and academic integrity. The focus of the future is to employ NLP resources responsibly into the education space to create equitable, personalized, and productive distributed learning environments.

## 6 Results and discussion

**Figure 8:**
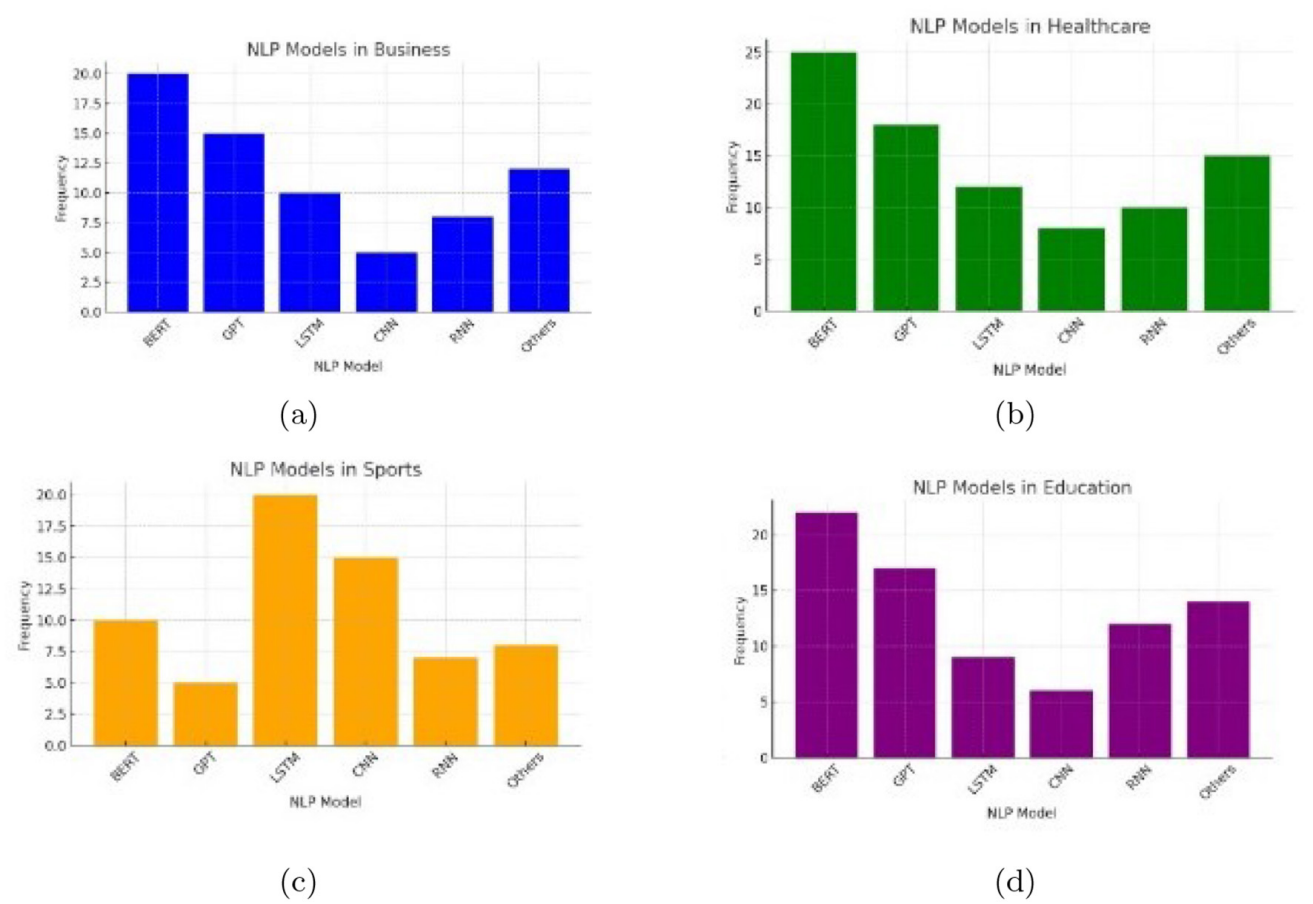
Histogram chart showing NLP model distribution in **(a)** business, **(b)** healthcare, **(c)** sports, **(d)** education.

**Figure 9:**
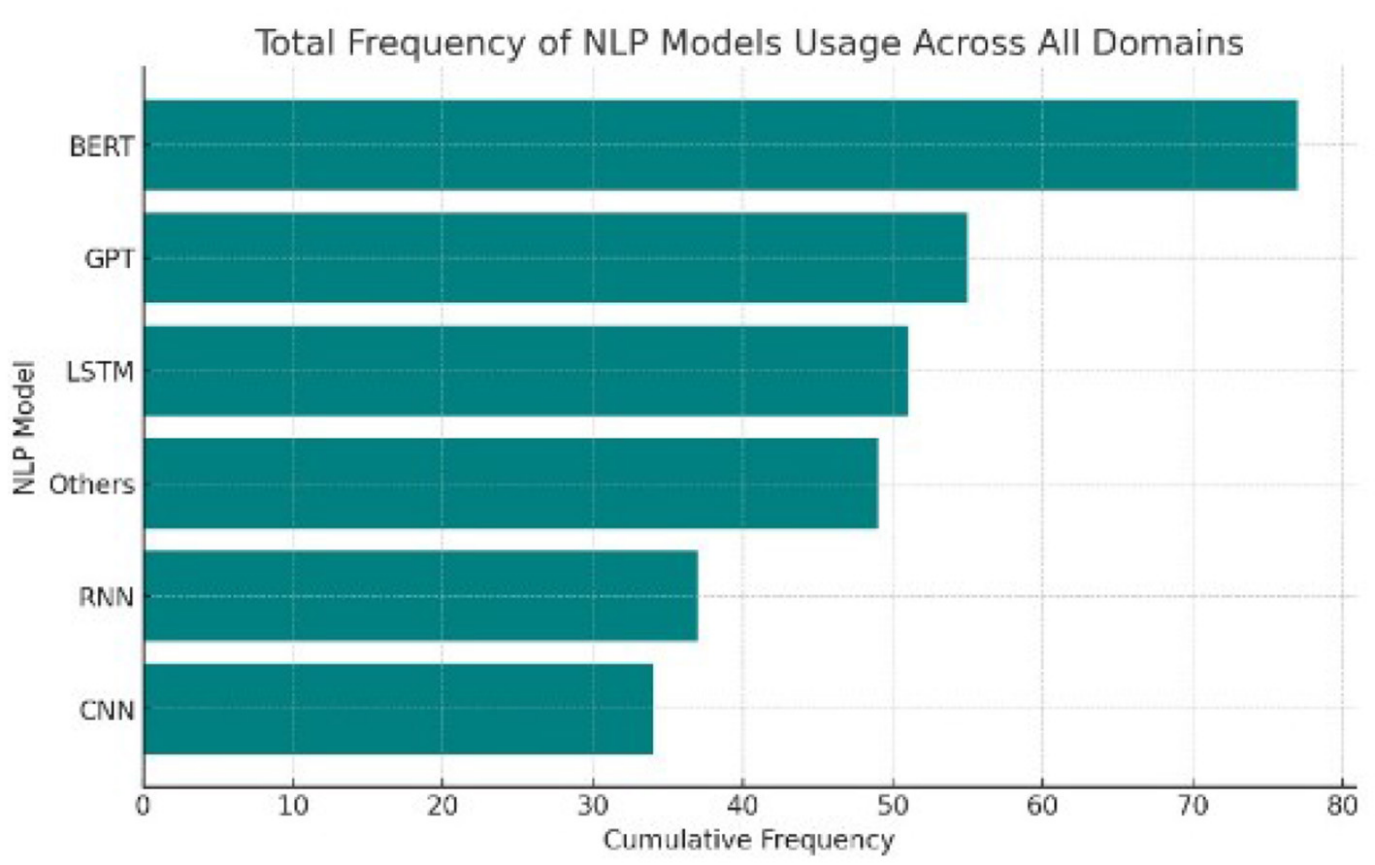
Cumulative analysis of different models used across all domains.

In the rapidly evolving field of Natural Language Processing (NLP), understanding how these different models are distributed and utilized across domains is vital. This analysis investigates the prevalence and performance of models like BERT, GPT, CNNs, and RNNs, thereby highlighting the specific strengths of each model as well as the particular environments to which each model responds. By analyzing histograms, cumulative frequency charts, pie charts, and heatmaps we can get valuable information about how models are used in different applications across healthcare, education, sports, and business. Figures 8, 9 explains the distribution of different methods in various application.

The histograms show how certain types of NLP models are used with differing usage across domains. For example, BERT is especially prevalent in the healthcare and education domains, which may only be because the model excels at complex language tasks such as processing medical text and assessments in education. As another example, CNNs or RNNs are used somewhat more in sports for their ability to handle sequential (or temporal) data to analyze performance or predict performance. Business applications seem to utilize some combination of NLP models for tasks such as sentiment analysis, business intelligence, and Customer Relationship Management. Such a mix of the most sophisticated contextual models with simpler approaches testifies to this need.

Through the cumulative frequency bar chart we can conclude that frequency of usage of the BERT and GPT is high across all domains, pointing toward general versatility and capability of BERT and GPT in a large area of NLP tasks. On the other hand, lower frequency usages are found within CNNs and RNNs, which probably are powerful but specialized for a slighter amount of applications. Strong “Others” also gives a hint that, apart from primary NLP tool utilization categories, many task-specific NLP models or tools are being used.

**Figure 10:**
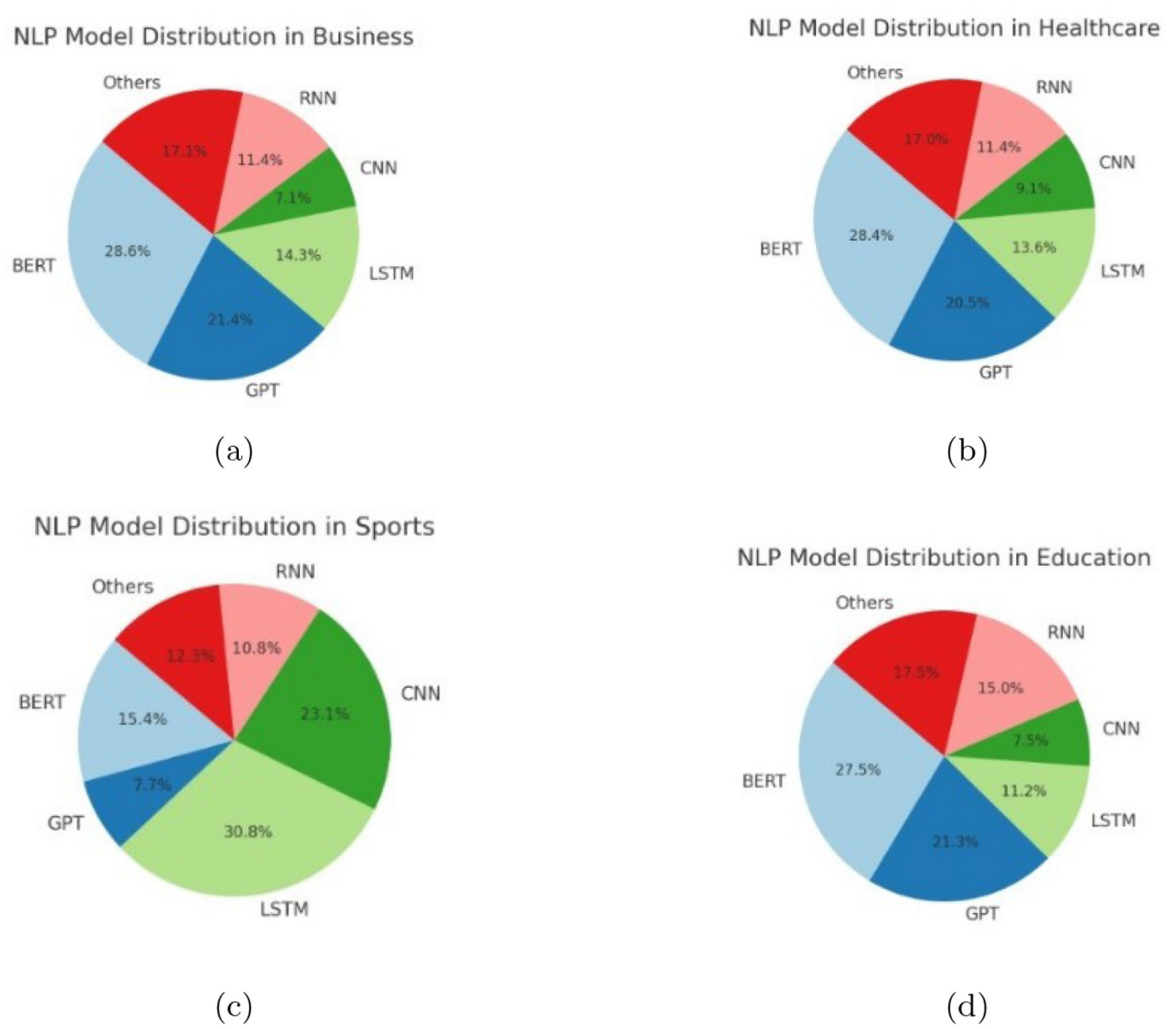
Pie chart showing NLP model distribution in **(a)** business, **(b)** healthcare, **(c)** sports, **(d)** education.

**Figure 11:**
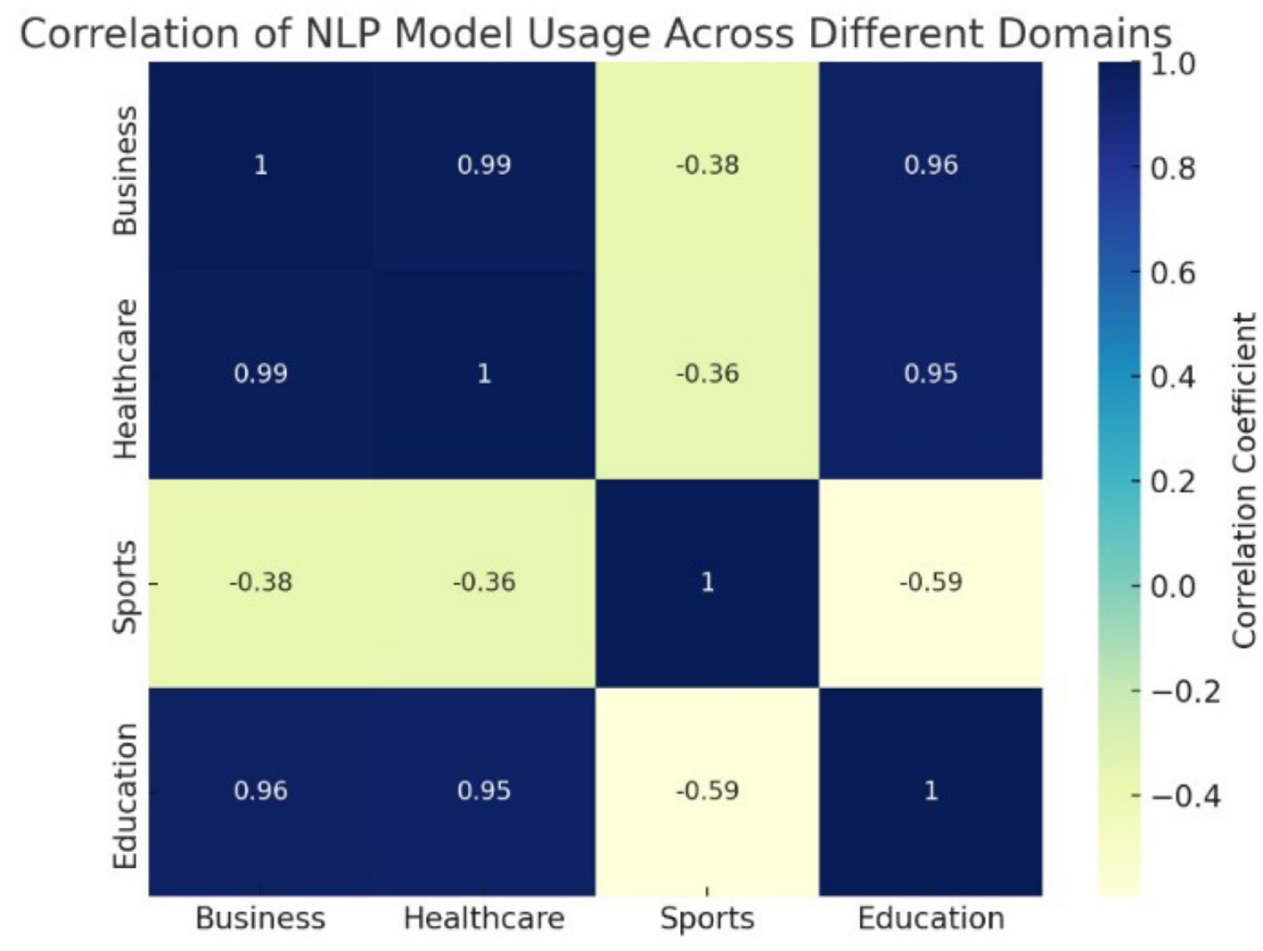
Heatmap of NLP models.

The pie charts, on the other hand, provide the share of NLP models in each domain. Healthcare has a significant share for such models as BERT and GPT, because they need powerful, contextually-enlightened models to clinically extract data and communicate with patients. Sports, however, have a better balance with an increased application of CNNs and LSTMs. That really proves to be of great importance when it comes to the analytics of dynamic data, for example, the movement of players and what was the final output of the game. Business usage is highly versatile. It mainly deals with sentiment analysis and making data-driven decisions through a chatbot approach. Figures 10, 11 shows the overall performance of the different methods.

Finally, the heatmap shows that there is considerable positive correlation between the usage of NLP models in healthcare and education models, likely because they share the need to extract and process heavy volumes of text data. There is rather weak correlation in the use of business and sports-related models, because business requires specific data structures that are not pertinent to sports. This indicates the presence of some across-domain applicability of the models, while other sectors may require sector-specific adaptations or altogether different types of models.

### 6.1 Model specialization vs. generalization

When looking at where and how NLP models are used across domains, it's helpful to consider the differences between generic models and models that support a particular use case.

- **Healthcare:** CNNs are often used for sequential text data as well as visual tasks, such as radiology and medical imaging. This indicates the duality of CNNs managing structured sequences and complex visual information, and text-heavy models (e.g. BERT and GPT) that can parse and analyze clinical texts or patient generated texts and documentation.
- **Sports:** Time-series modeling to understand player motion and game events (such as a player starting to shoot, throw a pass or a catch could be important events) performance prediction. CNNs and specifically recurrent neural networks (RNN) also long short-term memory networks (LSTM) play an important role modeling time-dependent patterns, and dynamic sequences which set sports apart from most fields that mainly analyze static or text-based artifacts.
- **Education:** BERT and GPT have been primarily used for text-based assessments, knowledge tracing, knowledge assessment, automated question-answer and summary tasking although have been evaluated in other modalities. For example (i.e., reading comprehension and document analysis).
- **Business:** Business applications use a variety of NLP models across sentiment analysis, chatbots, customer support, and business intelligence tasks. Using this combination reflects their need for both general-purpose models (text understanding) and dedicated (structured data and information that drives decision making) models.
- **Cross-Domain Synthesis:** On the whole, BERT and GPT show wide general-purpose applicability across a range of domains due to their strong contextual capabilities. LSTMs and CNNs are more specialized, as they do best in [(e.g., crypto-bottom) domains that require temporal modeling (LSTMs), or visual analysis (CNNs)]. Understanding the better application, both general and specialized, is important for undertaking modeling decision making based on task type or domain specific requirements.
- **Versatility vs. Specialization:** General-purpose models, such as BERT and GPT, can be generalized across domains, while specialized models such as CNNs/LSTMs work very well in task-specific scenarios.
- **Healthcare-Education Overlap:** The higher the overlap is, the greater the need to process textual data in a similar way.
- **Domain-Specific Requirements:** The cases of Sports and Business are domain-specific and hence require specialized modeling.
- **Task-Relevant Model Selection:** The choice of model is influenced by the type of data, whether text, visual, or temporal, and also by the application context.

## 7 Conclusion

Looking at how NLP is used in business, healthcare, sports, and education, it's clear it's changing things up everywhere. Businesses use it for customer stuff, figuring out feelings, and making things automatic. Healthcare uses it to pull data from patient info, help find problems, and talk to people. In sports, NLP helps break down how people play, get fans involved, and see tactics. And in education, it helps make learning personal, grades tests automatically, and makes tools that reach everyone.

New models like BERT and GPT are great at understanding context and can do a lot of things, while CNNs and LSTMs are better for things that happen in order. The way these models are used shows that people want NLP to adjust to what they need: complex models for healthcare and education, and quick and easy setups for business.

What's really important is that NLP is shaking up industries to help with better choices, faster work, and more involvement. As we move on, we should work on making it correct, new, and morally sound, including keeping information secret, cutting down on unfairness, and using it correctly everywhere.

In the days to come, we need to look into how NLP can be used in different fields, understand how the models work, and find ways to keep it from being used the wrong way. When experts in different fields work with AI folks, we can be sure NLP keeps making things better, fairer, and more trustworthy in all sorts of areas.

## Data Availability

The original contributions presented in the study are included in the article/supplementary material, further inquiries can be directed to the corresponding author.

## References

[B1] Abbasi N. (2024). Artificial intelligence in remote monitoring and telemedicine. J. Artif. Intellig. General Sci. 1, 258–272. doi: 10.60087/jaigs.v1i1.202

[B2] Adarsh R. Pillai R. H. Krishnamurthy A. Bi A. (2023). “Innovative business research in finance and marketing system based on ethically governed artificial intelligence,” in 2023 Eighth International Conference on Science Technology Engineering and Mathematics (ICONSTEM) (Chennai: IEEE), 1–8.

[B3] Al-Garadi M. A. Yang Y. C. Sarker A. (2022). The role of natural language processing during the COVID-19 pandemic: health applications, opportunities, and challenges. Healthcare 10:2270). doi: 10.3390/healthcare10112270 36421593 PMC9690240

[B4] Alowais S. A. Alghamdi S. S. Alsuhebany N. Alqahtani T. Alshaya A. I. Almohareb S. N. . (2023). Revolutionizing healthcare: the role of artificial intelligence in clinical practice. BMC Med. Educ. 23:689. doi: 10.1186/s12909-023-04698-z 37740191 PMC10517477

[B5] Alqahtani T. Badreldin H. A. Alrashed M. Alshaya A. I. Alghamdi S. S. bin Saleh K. . (2023). The emergent role of artificial intelligence, natural learning processing, and large language models in higher education and research. Res. Soc. Admin. Pharm. 19, 1236–1242. doi: 10.1016/j.sapharm.2023.05.016 37321925

[B6] Arnaud É. Elbattah M. Gignon M. Dequen G. (2021). “NLP-based prediction of medical specialties at hospital admission using triage notes,” in 2021 IEEE 9th International Conference on Healthcare Informatics (ICHI) (Victoria, BC: IEEE), 548–553.

[B7] Bahja M. (2020). Natural Language Processing Applications in Business.

[B8] Basyal G. P. Rimal B. P. Zeng D. (2020). A systematic review of natural language processing for knowledge management in healthcare. arXiv [preprint] arXiv:2007.09134. doi: 10.5121/csit.2020.100921

[B9] Beltagy I. Lo K. Cohan A. (2019). SciBERT: A pre-trained language model for scientific text. arXiv [preprint] arXiv:1903.10676. doi: 10.18653/v1/D19-1371

[B10] Berisha-Shaqiri A. (2014). Impact of information technology and the internet in businesses. Inform. Technol. 1, 73–79. doi: 10.5901/mjss.2015.v6n6p78

[B11] Bharadiya J. P. (2023). A comparative study of business intelligence and artificial intelligence with big data analytics. Am. J. Artif. Intellig. 7, 24–30. doi: 10.9734/jerr/2023/v25i3893

[B12] Bhutoria A. (2022). Personalized education and artificial intelligence in the United States, China, and India: a systematic review using a human-in-the-loop model. Comp. Educ.: Artif. Intellig. 3, 100068. doi: 10.1016/j.caeai.2022.100068

[B13] Bordignon A. C. D. A. Thom L. H. Silva T. S. Dani V. S. Fantinato M. Ferreira R. C. B. (2018). Natural language processing in business process identification and modeling: a systematic literature review. Simpósio Brasileiro de Sistemas de Informação (SBSI).

[B14] Bunker R. Susnjak T. (2022). The application of machine learning techniques for predicting match results in team sport: a review. J. Artif. Intellig. Res. 73, 1285–1322. doi: 10.1613/jair.1.13509

[B15] Burney S. A. Mahmood N. Abbas Z. (2010). Information and communication technology in healthcare management systems: prospects for developing countries. Int. J. Comp. Appl. 4, 27–32. doi: 10.5120/801-1138

[B16] Crossley S. Liu R. McNamara D. (2017). “Predicting math performance using natural language processing tools,” in Proceedings of the Seventh International Learning Analytics & *Knowledge Conference*, 339–347.

[B17] Crossley S. A. Kim M. Allen L. McNamara D. (2019). “Automated summarization evaluation (ASE) using natural language processing tools,” in Artificial Intelligence in Education: 20th International Conference, AIED 2019 (Chicago, IL: Springer International Publishing), 84–95.

[B18] Crowston K. Allen E. E. Heckman R. (2012). Using natural language processing technology for qualitative data analysis. Int. J. Soc. Res. Methodol. 15, 523–543. doi: 10.1080/13645579.2011.625764

[B19] Devaul H. Diekema A. R. Ostwald J. (2011). Computer-assisted assignment of educational standards using natural language processing. J. Am. Soc. Inform. Sci. Technol. 62, 395–405. doi: 10.1002/asi.21437

[B20] Dhivyashree M. Sarumathi K. R. Sabarmathi K. R. (2021). “A combined model of NLP with business process modelling for sentiment analysis,” in 2021 5th International Conference on Electronics, Communication and Aerospace Technology (ICECA) (Coimbatore: IEEE), 1308–1313.

[B21] Elbattah M. Arnaud É. Gignon M. Dequen G. (2021). The role of text analytics in healthcare: a review of recent developments and applications. Healthinf 2021, 825–832. doi: 10.5220/0010414508250832

[B22] Elmanaseer S. Rand A. (2023). “A proposed technique for business process modeling diagram using natural language processing,” in International Conference on Information Technology (ICIT).

[B23] Emad A. Bassel F. Refaat M. Abdelhamed M. Shorim N. AbdelRaouf A. (2021). “Automatic video summarization with timestamps using natural language processing text fusion,” in 2021 IEEE 11th Annual Computing and Communication Workshop and Conference (CCWC) (Las Vegas, NV: IEEE), 0060–0066.

[B24] Ferrari A. Spagnolo G. O. Fiscella A. Parente G. (2019). “QuOD: an NLP tool to improve the quality of business process descriptions,” in From Software Engineering to Formal Methods and Tools, and Back: Essays Dedicated to Stefania Gnesi on the Occasion of Her 65th Birthday (Cham: Springer International Publishing), 267–281.

[B25] Godbole M. Agarwal A. Sahay B. (2021). application of ai/ml/nlp technology into the business process modelling. Int. J. Adv. Res. Eng. Technol. (IJARET) 12, 37–50.

[B26] Goksel N. Bozkurt A. (2019). “Artificial intelligence in education: Current insights and future perspectives,” in Handbook of Research on Learning in the Age of Transhumanism (New York: IGI Global), 224–236.

[B27] Gupta P. Narang B. (2012). Role of text mining in business intelligence. Gian Jyoti E-J. 1, 1–13.

[B28] Han X. Dang Y. Mei L. Wang Y. Li S. Zhou X. (2019). “A novel part of speech tagging framework for nlp based business process management,” in 2019 IEEE International Conference on Web Services (ICWS) (Milan: IEEE), 383–387.

[B29] Heaton C. Prasenjit M. (2022). “Using machine learning to describe how players impact the game in the mlb,” in The 16th Annual MIT Sloan Sports Analytics Conference.

[B30] Hossain E. Rana R. Higgins N. Soar J. Barua P. D. Pisani A. R. . (2023). Natural language processing in electronic health records in relation to healthcare decision-making: a systematic review. Comput. Biol. Med. 155:106649. doi: 10.1016/j.compbiomed.2023.106649 36805219

[B31] Hua Y. Liu F. Yang K. Li Z. Na H. Sheu Y. H. . (2024). Large language models in mental health care: a scoping review. arXiv [preprint] arXiv:2401.02984. doi: 10.2196/preprints.64088

[B32] Ivanchikj A. Serbout S. Pautasso C. (2020). “From text to visual BPMN process models: design and evaluation,” in Proceedings of the 23rd ACM/IEEE International Conference on Model Driven Engineering Languages and Systems, 229–239.

[B33] Javaid M. Haleem A. Singh R. P. Khan S. Khan I. H. (2023). Unlocking the opportunities through ChatGPT tool towards ameliorating the education system. BenchCouncil Trans. Benchm. Stand. Eval. 3:100115. doi: 10.1016/j.tbench.2023.100115

[B34] Jose Ramon S. Ribeiro-Soriano D. Salda na P. (2022). Exploring the challenges of remote work on Twitter users' sentiments: from digital technology development to a post-pandemic era. J. Busin. Res. 142, 242–254. doi: 10.1016/j.jbusres.2021.12.052

[B35] Kang Y. Cai Z. Tan C.-W. Huang Q. Liu H. (2020). Natural language processing (NLP) in management research: A literature review. J. Managem. Analyt. 7, 1–34. doi: 10.1080/23270012.2020.1756939

[B36] Kerlyl A. Hall P. Bull S. (2006). “Bringing chatbots into education: Towards natural language negotiation of open learner models,” in International Conference on Innovative Techniques and Applications of Artificial Intelligence (London: Springer London), 179–192.

[B37] Khurana D. Koli A. Khatter K. Singh S. (2023). Natural language processing: state of the art, current trends and challenges. Multimed. Tools Appl. 82, 3713–3744. doi: 10.1007/s11042-022-13428-4 35855771 PMC9281254

[B38] Koroteev M. V. (2021). BERT: a review of applications in natural language processing and understanding. arXiv [preprint] arXiv:2103.11943. doi: 10.48550/arXiv.2103.11943

[B39] Lea C. Flynn M. D. Vidal R. Reiter A. Hager G. D. (2017). “Temporal convolutional networks for action segmentation and detection,” in Proceedings of the IEEE Conference on Computer Vision and Pattern Recognition (Honolulu, HI: IEEE), 156–165.

[B40] Lee E. E. Torous J. De Choudhury M. Depp C. A. Graham S. A. Kim H. C. . (2021). Artificial intelligence for mental health care: clinical applications, barriers, facilitators, and artificial wisdom. Biol. Psychiat.: Cognit. Neurosci. Neuroimag. 6, 856–864. doi: 10.1016/j.bpsc.2021.02.001 33571718 PMC8349367

[B41] Lee H. S. Lee J. (2021). Applying artificial intelligence in physical education and future perspectives. Sustainability 13:351. doi: 10.3390/su13010351

[B42] Leslie D. Mazumder A. Peppin A. Wolters M. K. Hagerty A. (2021). Does “AI” stand for augmenting inequality in the era of covid-19 healthcare? BMJ 372:n304. doi: 10.1136/bmj.n304 33722847 PMC7958301

[B43] Li M. Han L. Ma S. (2023). “Measuring sports teaching activities in higher education through natural language processing,” in Second International Conference on Statistics, Applied Mathematics, and Computing Science (CSAMCS 2022), 425–432.

[B44] Lucy L. Demszky D. Bromley P. Jurafsky D. (2020). Content analysis of textbooks via natural language processing: findings on gender, race, and ethnicity in Texas US history textbooks. AERA Open 6:2332858420940312. doi: 10.1177/2332858420940312

[B45] Lund B. D. Wang T. Mannuru N. R. Nie B. Shimray S. Wang Z. (2023). ChatGPT and a new academic reality: Artificial Intelligence-written research papers and the ethics of the large language models in scholarly publishing. J. Assoc. Inform. Sci. Technol. 74, 570–581. doi: 10.1002/asi.24750

[B46] Mah P. M. Skalna I. Muzam J. (2022). Natural language processing and artificial intelligence for enterprise management in the era of industry 4.0. Appl. Sci. 12:9207. doi: 10.3390/app12189207

[B47] Malinka K. Peresíni M. Firc A. Hujnák O. Janus F. (2023). “On the educational impact of ChatGPT: Is artificial intelligence ready to obtain a university degree?,” in Proceedings of the 2023 Conference on Innovation and Technology in Computer Science Education V. 1, 47–53.

[B48] Melton G. B. Hripcsak G. (2005). Automated detection of adverse events using natural language processing of discharge summaries. J. Am. Med. Inform. Assoc. 12, 448–457. doi: 10.1197/jamia.M1794 15802475 PMC1174890

[B49] Michaud L. N. McCoy K. F. (1999). “Modeling user language proficiency in a writing tutor for deaf learners of English,” in Computer Mediated Language Assessment and Evaluation in Natural Language Processing.

[B50] Michaud L. N. McCoy K. F. (2006). Capturing the evolution of grammatical knowledge in a CALL system for deaf learners of English. Int. J. Artif. Intellig. Educ. 16, 65–97. doi: 10.3233/IRG-2006-16(1)04

[B51] Mijwil M. Aljanabi M. Ali A. H. (2023). Chatgpt: Exploring the role of cybersecurity in the protection of medical information. Mesopotam. J. cybersecurity 2023, 18–21. doi: 10.58496/MJCS/2023/004

[B52] Mishra A. Soni U. Arunkumar A. Huang J. Kwon B. C. Bryan C. (2023). Promptaid: Prompt exploration, perturbation, testing and iteration using visual analytics for large language models. arXiv [preprint] arXiv:2304.01964. doi: 10.48550/arXiv.2304.01964 40031291

[B53] Moon N. N. Salehin I. Parvin M. Hasan M. M. Talha I. M. Debnath S. C. . (2021). Natural language processing based advanced method of unnecessary video detection. Int. J. Elect. Comp. Eng. 11, 5411–5419. doi: 10.11591/ijece.v11i6.pp5411-5419

[B54] Mukherjee S. Shekhar S. Martinez S. S. Gupta A. K. Kumar S. Karrar A. Z. (2023). “Transforming operations data into business intelligence: leveraging Natural Language Processing (NLP) and Machine Learning (ML) for accurate and sustainable insights,” in Abu Dhabi International Petroleum Exhibition and Conference (Abu Dhabi: SPE).

[B55] Mustansir A. Shahzad K. Malik M. K. (2022). Towards automatic business process redesign: an NLP based approach to extract redesign suggestions. Autom. Softw. Eng. 29:1. doi: 10.1007/s10515-021-00316-8

[B56] Nagaraj P. Muneeswaran V. Rohith B. Vasanth B. S. Reddy G. V. V. Teja A. K. (2023). “Automated youtube video transcription to summarized text using natural language processing,” in 2023 International Conference on Computer Communication and Informatics (ICCCI) (Coimbatore: IEEE), 1–6.

[B57] Nazi Z. A. Peng W. (2024). Large language models in healthcare and medical domain: a review. Informatics 11:57. doi: 10.3390/informatics11030057

[B58] Olujimi P. A. Ade-Ibijola A. (2023). NLP techniques for automating responses to customer queries: a systematic review. Discover Artif. Intellig. 3:20. doi: 10.1007/s44163-023-00065-5

[B59] Park E. H. Watson H. I. Mehendale F. V. O'Neil A. Q. (2022). Evaluating the impact on clinical task efficiency of a natural language processing algorithm for searching medical documents: Prospective crossover study. JMIR Med. Inform. 10:e39616. doi: 10.2196/39616 36287591 PMC9647457

[B60] Pavitt J. Braines D. Tomsett R. (2021). Cognitive analysis in sports: supporting match analysis and scouting through artificial intelligence. Appl. AI Letters 2, e21. doi: 10.1002/ail2.21

[B61] Raiaan M. A. K. Mukta M. S. H. Fatema K. Fahad K. Sakib S. Mim M. M. J. (2024). A review on large language models: architectures, applications, taxonomies, open issues and challenges. IEEE Access 12, 26839–26874. doi: 10.1109/ACCESS.2024.3365742

[B62] Rathore B. (2023). Digital Transformation 4.0: Integration of Artificial Intelligence & *Metaverse in Marketing*.

[B63] Reeves R. M. Christensen L. Brown J. R. Conway M. Levis M. Gobbel G. T. . (2021). Adaptation of an NLP system to a new healthcare environment to identify social determinants of health. J. Biomed. Inform. 120:103851. doi: 10.1016/j.jbi.2021.103851 34174396 PMC8386129

[B64] Reshamwala A. Mishra D. Pawar P. (2013). Review on natural language processing. *IRACST Eng*. Sci. Technol. Int. J. 3, 113–116.

[B65] Salas-Pilco S. Z. Yang Y. (2022). Artificial intelligence applications in Latin American higher education: a systematic review. Int. J. Educ. Technol. Higher Educ. 19:21. doi: 10.1186/s41239-022-00326-w PMC9111674 35600418

[B66] Schönfelder P. König M. (2021). “Deep learning-based entity recognition in construction regulatory documents,” in ISARC. Proceedings of the International Symposium on Automation and Robotics in Construction, Vol. 38 (IAARC Publications), 387–394.

[B67] Shen J. T. Yamashita M. Prihar E. Heffernan N. Wu X. Graff B. . (2021). MathBERT: A pre-trained language model for general NLP tasks in mathematics education. arXiv [preprint] arXiv:2106.07340. doi: 10.48550/arXiv.2106.07340

[B68] Sinha R. K. Roy A. D. Kumar N. Mondal H. (2023). Applicability of ChatGPT in assisting to solve higher order problems in pathology. Cureus 15:35237. doi: 10.7759/cureus.35237 36968864 PMC10033699

[B69] Sintoris K. Vergidis K. (2017). “Extracting Business Process Models Using Natural Language Processing (NLP) Techniques,” in 2017 IEEE 19th Conference on Business Informatics (CBI) (Thessaloniki: IEEE), 135–139.

[B70] Sousa G. (2022). Natural Language Processing and its applications in e-business. Cadernos de Investigação do Mestrado em Negócio Eletrónico 2:2. doi: 10.56002/ceos.0070_cimne_1_2

[B71] Subrahmanya S. V. G. Shetty D. K. Patil V. Hameed B. Z. Paul R. Smriti K. . (2022). The role of data science in healthcare advancements: applications, benefits, and future prospects. Irish J. Med. Sci. 191, 1473–1483. doi: 10.1007/s11845-021-02730-z 34398394 PMC9308575

[B72] Tayefi M. Ngo P. Chomutare T. Dalianis H. Salvi E. Budrionis A. . (2021). Challenges and opportunities beyond structured data in analysis of electronic health records. Wiley Interdiscipl. Rev.: Comp. Stat. 13:e1549. doi: 10.1002/wics.1549

[B73] Taymouri F. Marcello La R. Marlon D. Fabrizio Maria M. (2021). Business process variant analysis: survey and classification. Knowl.-Based Syst. 211:106557. doi: 10.1016/j.knosys.2020.106557

[B74] Tvardik N. Kergourlay I. Bittar A. Segond F. Darmoni S. Metzger M. H. (2018). Accuracy of using natural language processing methods for identifying healthcare-associated infections. Int. J. Med. Inform. 117, 96–102. doi: 10.1016/j.ijmedinf.2018.06.002 30032970

[B75] Wanless L. Seifried C. Bouchet A. Valeant A. Naraine M. L. (2022). The diffusion of natural language processing in professional sport. Sport Managem. Rev. 25, 522–545. doi: 10.1080/14413523.2021.1968174

[B76] Weissbock J. Inkpen D. (2014). “Combining textual pre-game reports and statistical data for predicting success in the national hockey league,” in Canadian Conference on Artificial Intelligence (Cham: Springer International Publishing), 251–262.

[B77] Xi X. Zhang C. Jia W. Jiang R. (2024). Enhancing human pose estimation in sports training: Integrating spatiotemporal transformer for improved accuracy and real-time performance. Alexandria Eng. J. 109, 144–156. doi: 10.1016/j.aej.2024.08.072

[B78] Xia H. Yang Z. Zhao Y. Wang Y. Li J. Tracy R. . (2024). Language and Multimodal Models in Sports: A Survey of Datasets and Applications.

[B79] Xu J. Glicksberg B. S. Su C. Walker P. Bian J. Wang F. (2021). Federated learning for healthcare informatics. J. Healthc. Inform. Res. 5, 1–19. doi: 10.1007/s41666-020-00082-4 33204939 PMC7659898

[B80] Xu Z. Zhu P. (2023). Using BERT-based textual analysis to design a smarter classroom mode for computer teaching in higher education institutions. Int. J. Emerg. Technol. Learn. (iJET) 18, 114–127. doi: 10.3991/ijet.v18i19.42483

[B81] Yamashita R. Nishio M. Do R. K. G. Togashi K. (2018). Convolutional neural networks: an overview and application in radiology. Insights Imag. 9, 611–629. doi: 10.1007/s13244-018-0639-9 29934920 PMC6108980

[B82] Yang R. Li Z. Tang H. Zhu K. (2022a). “ChatMatch: evaluating chatbots by autonomous chat tournaments,” in Proceedings of the 60th Annual Meeting of the Association for Computational Linguistics (Dublin: Association for Computational Linguistics), 7579–7590.

[B83] Yang S. J. Ogata H. Matsui T. Chen N. S. (2021). Human-centered artificial intelligence in education: Seeing the invisible through the visible. Comp. Educ.: Artif. Intellig. 2:100008. doi: 10.1016/j.caeai.2021.100008

[B84] Yang X. Chen A. PourNejatian N. Shin H. C. Smith K. E. Parisien C. . (2022b). A large language model for electronic health records. NPJ Digit. Med. 5:194. doi: 10.1038/s41746-022-00742-2 36572766 PMC9792464

[B85] Yang Z. Xia H. Li J. Chen Z. Zhu Z. Shen W. (2024). Sports intelligence: assessing the sports understanding capabilities of language models through question answering from text to video. arXiv [preprint] arXiv:2406.14877. doi: 10.3390/electronics14030461

[B86] Zhai X. Chu X. Chai C. S. Jong M. S. Y. Istenic A. Spector M. . (2021). A review of artificial intelligence (AI) in education from 2010 to 2020. Complexity 2021:8812542. doi: 10.1155/2021/8812542

[B87] Zhang A. Xing L. Zou J. Wu J. C. (2022). Shifting machine learning for healthcare from development to deployment and from models to data. Nat. Biomed. Eng. 6, 1330–1345. doi: 10.1038/s41551-022-00898-y 35788685 PMC12063568

[B88] Zhang F. Fleyeh H. Wang X. Lu M. (2019). Construction site accident analysis using text mining and natural language processing techniques. Autom. Construct. 99, 238–248. doi: 10.1016/j.autcon.2018.12.016

[B89] Zhang G. Fan Y. (2024). Application of natural language processing to the development of sports biomechanics in China: a literature review of journal abstracts in Chinese between 1980 and 2022. Kinesiol. Rev. 1, 1–15. doi: 10.1123/kr.2023-0038

[B90] Zhang J. Han D. Han S. Li H. Lam W. K. Zhang M. (2025). ChatMatch: Exploring the potential of hybrid vision-language deep learning approach for the intelligent analysis and inference of racket sports. Comp. Speech & *Lang*. 89:101694. doi: 10.1016/j.csl.2024.101694

[B91] Zhou B. Yang G. Shi Z. Ma S. (2022). Natural language processing for smart healthcare. IEEE Rev. Biomed. Eng. 17, 4–18. doi: 10.1109/RBME.2022.3210270 36170385

